# Unseen Enemy: Mechanisms of Multidrug Antimicrobial Resistance in Gram-Negative ESKAPE Pathogens

**DOI:** 10.3390/antibiotics14010063

**Published:** 2025-01-09

**Authors:** Giedrė Valdonė Sakalauskienė, Lina Malcienė, Edgaras Stankevičius, Aurelija Radzevičienė

**Affiliations:** Institute of Physiology and Pharmacology, Faculty of Medicine, Medical Academy, Lithuanian University of Health Sciences, 44307 Kaunas, Lithuania; lina.malciene@lsmu.lt (L.M.); edgaras.stankevicius@lsmu.lt (E.S.); aurelija.radzeviciene@lsmu.lt (A.R.)

**Keywords:** antimicrobial resistance, multidrug-resistant gram-negative ESKAPE pathogens, mechanisms of antimicrobial resistance

## Abstract

Multidrug antimicrobial resistance (AMR) represents a formidable challenge in the therapy of infectious diseases, triggered by the particularly concerning gram-negative *Enterococcus faecium*, *Staphylococcus aureus*, *Klebsiella pneumoniae*, *Acinetobacter baumannii*, *Pseudomonas aeruginosa*, and *Enterobacter* spp. (ESKAPE) pathogens. Designated as a “priority” in 2017, these bacteria continue to pose a significant threat in 2024, particularly during the worldwide SARS-CoV-2 pandemic, where coinfections with ESKAPE members contributed to worsened patient outcomes. The declining effectiveness of current treatments against these pathogens has led to an increased disease burden and an increase in mortality rates globally. This review explores the sophisticated mechanisms driving AMR in gram-negative ESKAPE bacteria, focusing on *Acinetobacter baumannii*, *Klebsiella pneumoniae*, *Pseudomonas aeruginosa*, and *Enterobacter* spp. Key bacterial mechanisms contributing to resistance include limitations in drug uptake, production of antibiotic-degrading enzymes, alterations in drug target sites, and enhanced drug efflux systems. Comprehending these pathways is vital for formulating innovative therapeutic strategies and tackling the ongoing threat posed by these resistant pathogens.

## 1. Introduction

**Figure 1:**
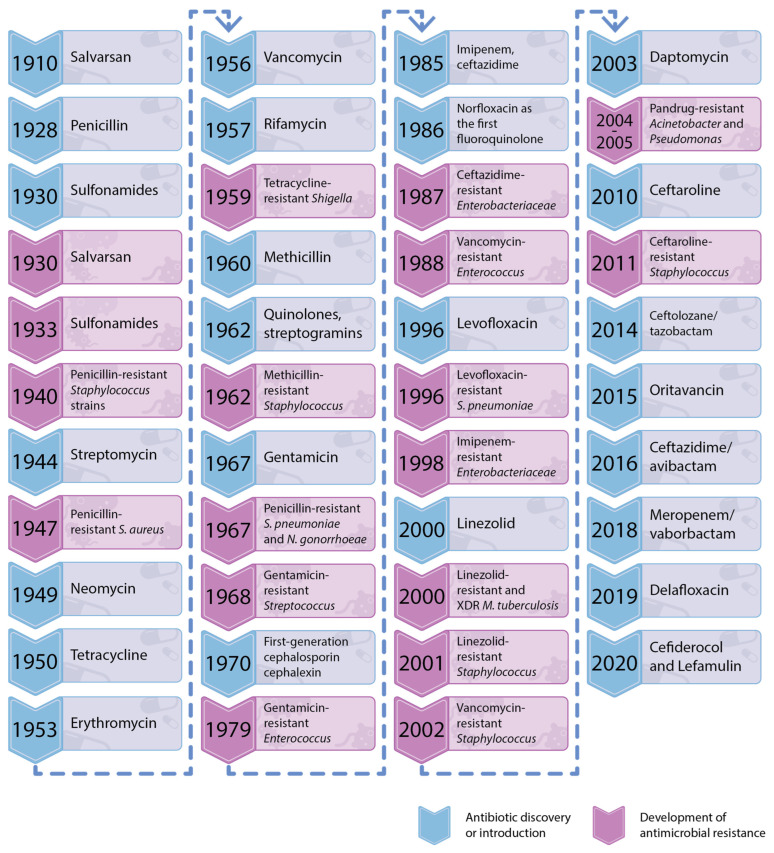
Progression of antibiotic discovery, clinical introduction, and antimicrobial resistance development (adapted and reused from the source [4] under the condition of the Creative Commons Attribution-Non-Commercial-Share Alike 3.0 License, also supplemented on the basis of data from the sources [3,6]).

In 1945, during his Nobel Prize acceptance lecture, Sir Alexander Fleming (1881–1955) issued a strong warning to the medical community: “It is not difficult to make microbes resistant to penicillin in the laboratory by exposing them to concentrations not sufficient to kill them, and the same thing has occasionally happened in the body. The time may come when penicillin can be bought by anyone in the shops. Then, there is the danger that the ignorant man may easily underdose himself and by exposing his microbes to non-lethal quantities of the drug make them resistant” [1]. Unfortunately, the warning has not been properly taken into consideration. Although multidrug antimicrobial resistance (AMR) is a natural and inevitable process, wide spread misuse and overuse of antimicrobial agents have provoked a rapid development of this phenomenon [2,3,4,5]. New classes of antibiotics and synthetic antibacterial agents were developed between 1940 and 1980, with particular intensity during the 1950s and the 1960s. Antibacterial drug development has faced a sharp decline in recent decades, parallelled by an alarming increase in drug-resistant pathogens (Figure 1) [3,4,5,6].

**Figure 2:**
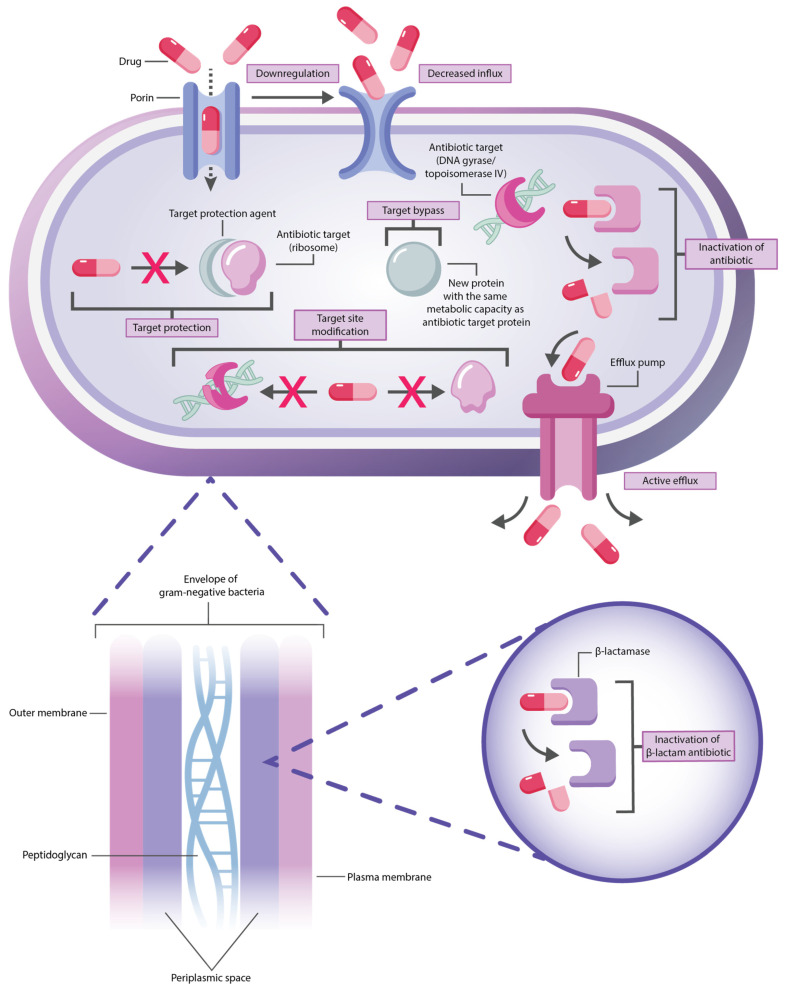
Bacterial mechanisms of AMR: structural and functional adaptation (reused from the source [14] under the condition of the Creative Commons CC BY license).

Thus, since the early 21st century, decades after the Golden Age of antimicrobials, bacterial infections have re-emerged as a significant challenge to public health [7,8]. The World Health Organization (WHO) has recently issued a warning that the world has been “running out of antibiotics” and AMR has been reaching a new peak [9,10]. Based on these facts, and due to growing concerns about its potential impact on global health—which may not always receive the attention it warrants—AMR is widely referred to as the “silent pandemic”. Throughout the COVID-19 crisis, reliance on broad-spectrum antibiotics without adequate diagnostic support became widespread, exacerbating the acceleration of antimicrobial resistance. Factors such as antibiotic overuse in agriculture, fewer novel drugs and vaccines in development, environmental toxicity, and limited funding for AMR research have compounded the issue. These practices drive structural and functional adaptations in bacteria, including enhanced efflux pumps, enzymatic modification, and biofilm formation, making infections harder to treat (Figure 2) [6,11,12,13,14].

This insidious spread of resistance increasingly impacts vulnerable populations, such as cancer patients, and poses a global health crisis that advances largely unchecked [6,11,12,13]. This article examines the mechanisms of antimicrobial resistance (AMR) specifically in gram-negative bacteria such as *A. baumannii*, *K. pneumoniae*, *P. aeruginosa*, and *Enterobacter* spp., which are part of the ESKAPE group (*E. faecium*, *S. aureus*, *K. pneumoniae*, *A. baumannii*, *P. aeruginosa*, and *Enterobacter* spp.) pathogens. These pathogens warrant significant attention due to their exceptional ability to acquire and develop resistance to multiple antibiotic classes. They are leading causes of nosocomial infections, including bloodstream infections, particularly in hospital settings, where their prevalence continues to challenge global health. Their complex resistance mechanisms, including intrinsic resistance (from protective outer membranes and efflux pumps) and acquired resistance (via chromosomal mutations and horizontal gene transfer), make infections caused by these bacteria increasingly challenging to treat [15,16,17]. Other AMR mechanisms, such as target site mutation and modification, are shared with gram-positive bacteria [14,18]. However, gram-negative ESKAPE bacteria often produce hydrolytic enzymes like β-lactamases that degrade critical antibiotics, further driving multidrug-resistant (MDR) strains (Figure 2) [15,16,17]. Thus, gram-negative ESKAPE bacteria exhibit a broader range of AMR mechanisms compared to gram-positive pathogens from this group [18].

The COVID-19 pandemic added new challenges to managing AMR, especially in intensive care units (ICUs). Many ICU patients required intubation, increasing their risk of developing ventilator-associated pneumonia (VAP), which is often caused by drug-resistant bacteria and is difficult to treat. This risk is particularly high with gram-negative pathogens such as *P. aeruginosa*, *Acinetobacter* spp., and *K. pneumoniae*, as well as with the gram-positive bacterium *S. aureus* [19,20,21].

Thus, the persistence of these MDR isolates in healthcare settings, both prior to and throughout the COVID-19 crisis, emphasizes the need for improved stewardship, ongoing surveillance, and innovative treatment strategies to reduce their impact [16,17,22].

Considering the alarming global situation regarding AMR, this article analyzes the contribution of gram-negative ESKAPE bacteria to AMR, focusing on the resistance mechanisms employed by these pathogens, including drug uptake limitation, drug target modification, enzymatic drug inactivation, and drug efflux, as well as the emergence of high-risk clones and novel treatment options.

## 2. The Worldwide Burden of AMR and the Critical Role of ESKAPE Bacteria

The global burden of a “silent pandemic” is enormous. In the United Kingdom, the number of individuals diagnosed with AMR-related infections rose from 61,946 in 2018 to 65,162 in 2019. In the European Union (EU) and European Economic Area (EEA), bacterial diseases were responsible for over 670,000 incidents annually, resulting in around 33,000 deaths directly attributed to these infections. In the Americas, which encompasses 35 countries, AMR was linked to approximately 569,000 deaths (95% UI: 406,000–771,000), with 141,000 deaths (99,900–196,000) directly caused by bacterial AMR [6,12,22]. In 2019, AMR was responsible for 1.27 million deaths and contributed to 4.95 million deaths globally. By 2050, it is anticipated that AMR could result in 10 million deaths per year worldwide [6,12,23,24]. Beyond its impact on mortality and health, AMR presents a significant economic strain. The World Bank has recently estimated that AMR could add US$1 trillion in healthcare costs by 2050 and lead to global GDP losses between US$1 trillion and US$3.4 trillion annually by 2030 [23,24]. AMR refers to the threatening phenomenon where pathogens such as bacteria, viruses, fungi, and parasites no longer respond to treatments they were once sensitive to, complicating the management of infections caused by these organisms. Bacteria are considered resistant if they stop responding to antibacterial agents and can grow, proliferate, and multiply under the exposure of inhibitory or bactericidal concentrations [2,5,9,23,24,25].

The most problematic microorganisms are multidrug-resistant (MDR), extensively drug-resistant (XDR), and pandrug-resistant (PDR) bacteria. MDR bacteria are resistant to multiple agents; XDR to nearly all drugs except colistin and tigecycline; and PDR to all antibacterial agents, including colistin and tigecycline. Classical resistant pathogens belong to the ESKAPE group, which includes *E. faecium*, methicillin-resistant *S. aureus*, *K. pneumoniae*, *A. baumannii*, *P. aeruginosa*, and *Enterobacter* spp. Currently, the most concerning among them are carbapenem-resistant Enterobacterales (CRE), carbapenem-resistant *K. pneumoniae* (CRKP), methicillin-resistant *S. aureus* (MRSA), ESBL-producing Enterobacterales, vancomycin-resistant *Enterococcus* (VRE), multidrug-resistant *P. aeruginosa*, and multidrug-resistant *Acinetobacter* [3,4,25,26,27].

**Figure 3:**
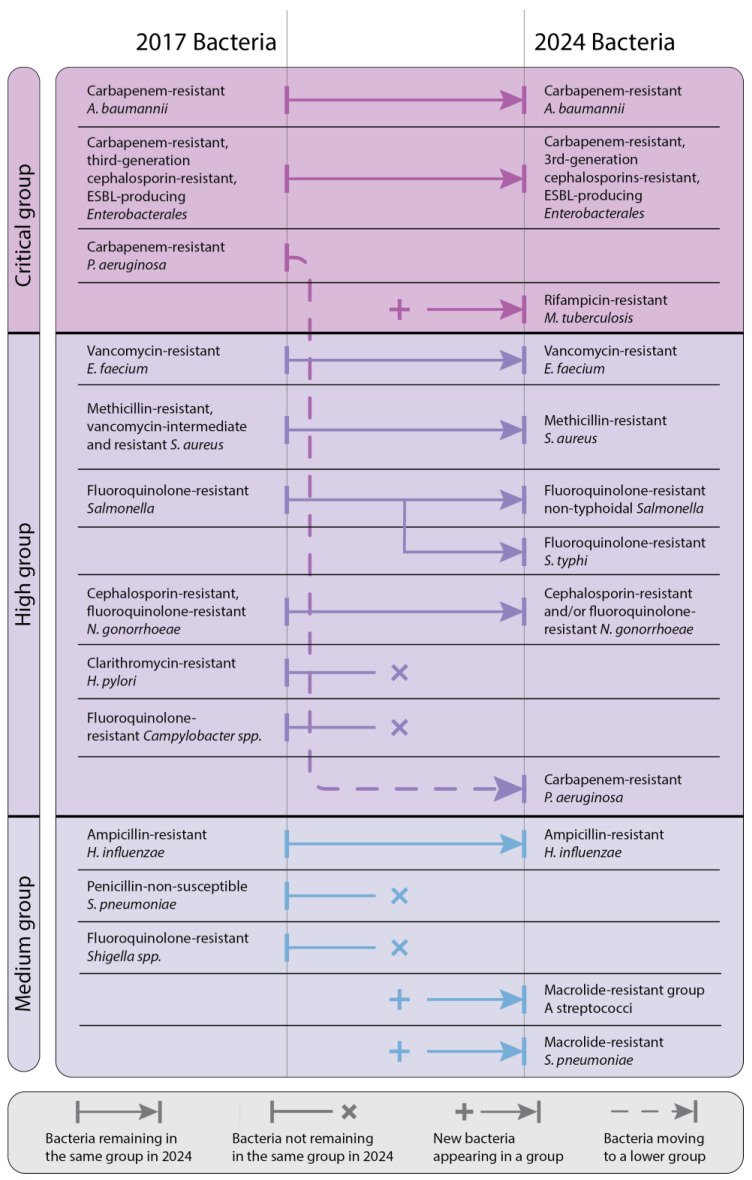
Changes in the WHO bacterial priority pathogens list during 2017–2024 (made on the basis of data from [3,4,6]).

Thus, in 2017, ESKAPE bacteria were designated as high-priority pathogens for the research and development of new antibacterial treatments aimed at countering them [2,27]. Next to the ESKAPE group, the WHO has identified other critically important bacteria for their resistance and clinical relevance. Significant changes in bacteria resistance have been noticed between 2017 and 2024 due to the dynamic nature of AMR (Figure 3) [28,29,30].

**Figure 4:**
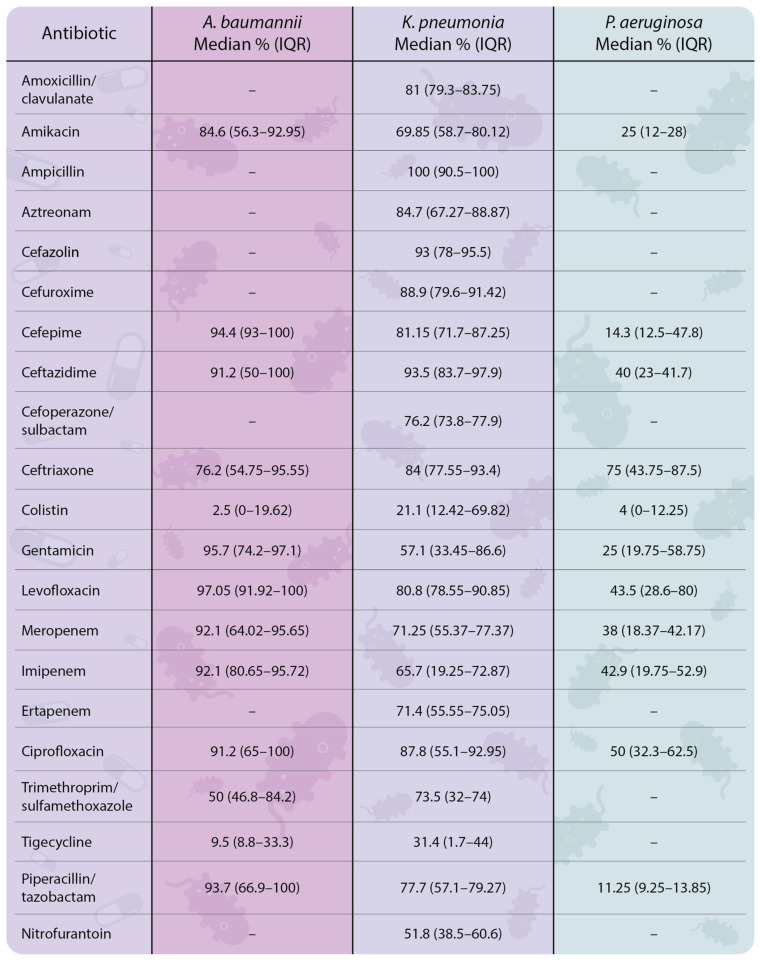
Antibiotic resistance profiles of gram-negative ESKAPE bacteria during the COVID-19 pandemic (adapted and reused from the source [19] under the condition of the Creative Commons CC BY license). Note: The picture reflects the data obtained from the aforementioned countries.

ESKAPE bacteria have become a worldwide menace during the SARS-CoV-2 pandemic. Soon after its declaration, half of the individuals with severe SARS-CoV-2 infection were found to have bacterial coinfections, mainly caused by pathogens from the ESKAPE group [18]. A systematic review analyzing data from 23 well-conducted studies published between 2019 and 2022 revealed high levels of AMR in *A. baumannii* and *K. pneumoniae*. The studies highlighting high levels of antimicrobial resistance were conducted across several countries, with the following distribution: four studies in India, four in Iran, two in Saudi Arabia, two in China, two in Turkey, two in Italy, and one each in Switzerland, USA, Serbia, Greece, Egypt, Pakistan, and Indonesia. Those aforementioned countries already had minor or moderate resistance rates, but during 2018–2020 reported an increased resistance to reserved antibiotics such as polymyxins, especially in India [31]. *A. baumannii* exhibited high resistance to levofloxacin (median [M] 97.05%; interquartile range [IQR] 91.92–100%), gentamicin (95.7%; 74.2–97.1%), cefepime (94.4%; 93–100%), and piperacillin/tazobactam (93.7%; 66.9–100%). For *K. pneumoniae*, the median of resistance to ampicillin, cefazolin, ceftazidime, and trimethoprim/sulfamethoxazole was 100% (IQR 90.5–100%), 93% (IQR 78–95.5%), 93.5% (IQR 83.7–97.9%), and 73.5% (IQR 32–74%), respectively. Although the overall AMR levels in *P. aeruginosa* were lower, resistance to ceftriaxone was observed at 75% (IQR 43.75–87.5%) (Figure 4).

Additionally, the SARS-CoV-2 pandemic contributed to a surge in the global use of personal protective devices and hygiene products, including soaps, handwashing solutions, and alcohol-based sanitizers, exacerbating the ongoing issue of AMR [26].

Despite the changes made in the WHO Bacterial Priority Pathogens List 2024, ESKAPE bacteria remain as a global alert, especially among critically ill and immunocompromised individuals [26,32]. These pathogens have reduced therapeutic choices for severe infections, increasing the severity of disease and mortality rates due to the acquisition of antimicrobial resistance genes [27]. For example, in 2017, a patient in the U.S. died from a *K. pneumoniae* strain resistant to every available antibiotic agent [33].

It has been established that ESKAPE pathogens have developed resistance to a wide range of antibiotics, including oxazolidinones, lipopeptides, macrolides, fluoroquinolones, tetracyclines, tigecycline, β-lactams, β-lactam/β-lactamase inhibitor combinations, carbapenems, glycopeptides, and polymyxins. However, resistance to lipoglycopeptides is less common and has only recently been observed [26,27]. Thus, to develop effective and targeted strategies to combat AMR of the ESKAPE group, the molecular mechanisms underlying this phenomenon must be fully clarified [34,35].

Extremely specific cellular components secreted across the plasma membrane have recently generated interest due to being the structures widely involved in the transmission of biological information, killing of competitive bacteria, and exerting drug resistance. These components are outer membrane vesicles (OMV) [36,37].

## 3. AMR Mechanisms in Gram-Negative ESKAPE Pathogens

### 3.1. A. baumannii

#### 3.1.1. Drug Uptake Limitation

β-barrel-shaped monomeric or trimeric porins are mainly presented in the outer membrane (OM) of gram-negative bacteria. These porins are called outer membrane proteins (Omps). Their function is to facilitate the diffusion of small molecules, such as nutrients and various antibiotics, into and out of the periplasmic space by creating tiny channels that allow for the passive transport of hydrophilic compounds and modulate cellular permeability [38,39].

Monomeric OmpA is the most expressed porin in the OM of *A. baumannii*. The OmpA porin is made up of an outer membrane β-barrel or N-terminal domain, as well as a periplasmic or C-terminal domain. While the N-terminal domain functions as transfer and shifts negatively charged β-lactams, such as β-lactamase inhibitor sulbactam and carbapenems like imipenem, into bacterial cells, the C-terminal domain interacts with peptidoglycan to support cell wall integrity and stability [40]. In wild strains, the C-terminal domain plays a key role in determining the sensitivity of *A. baumannii* to antibiotics such as nalidixic acid, chloramphenicol, the monobactam aztreonam, and the carbapenems imipenem and meropenem [38].

However, in mutant *A. baumannii* strains, due to the mutation in the *ompA* gene, the minimal inhibitory concentrations (MICs) of antibiotics, including nalidixic acid, chloramphenicol, trimethoprim, aztreonam, imipenem, meropenem as well as colistin are reduced. These findings indicate that additional mechanisms may contribute to a reduced susceptibility to these antibiotics, likely involving the expression of efflux pumps. Although the precise mechanism remains unclear, OmpA may be involved in the removal of compounds from the periplasmic space across the OM, potentially working in conjunction with the inner membrane (IM) efflux systems, such as the major facilitator family (MFS) pumps or resistance-nodulation-division (RND) systems that lack an Omp component [41].

It has been also found that in mutants, permeability of cephalothin/cephaloridine is 2- to 3-fold reduced compared to the wild strains of *A. baumannii* due to alterations in OmpA [39]. Bacteria can also regulate the production of OMV via the interaction of the periplasmic domain of OmpA with peptidoglycan and preserve the stability of the membrane [39]. It has been established that OmpA plays a role in biofilm formation and contributes to the adhesion of abiotic surfaces as well as to cells of various lineages, such as epithelial cells. OmpA acts as a virulence factor, significantly contributing to the pathogenicity of *A. baumannii* and stimulating the innate immune response [38,40,41,42]. Other porins detected in *A. baumannii* are CarO, OprD, OprC, OprW, OprF, Omp 22–23 kDa, Omp 33–36 kDa, Omp 37 kDa, Omp 43 kDa, Omp 44 kDa, Omp 47 kDa, AbuO, TolB, DcaP, Oma87/BamA, NmRmpM, CadF, and CarOd. Many of them are the leading cause of resistance to carbapenems, especially CarO protein encoded by the *CarO* gene [38,43].

Alterations in the *CarO* gene may lead to significant structural modifications in the CarO porin, resulting in reduced OM permeability and the emergence of drug resistance. A complete loss of the CarO porin leads to bacterial insensitivity to imipenem and meropenem [32,44]. Thus, the synthesis of genetically altered porins that diminish OM permeability or decrease the expression of these proteins is primarily linked to β-lactam-related AMR, while the mechanism of reduced permeability is less significant for other antibiotics, such as fluoroquinolones [2,44].

#### 3.1.2. Drug Target Modification

Lipopolysaccharides (LPSs) are the core components of the OM in the cells of *A. baumannii*. This component is targeted by polypeptide antibiotics such as colistin, a critical treatment option for severe infections induced by MDR bacteria, including *A. baumannii* [38,45]. The main two mechanisms of AMR involve alterations to the LPS complex and structural changes in lipid A, as well as the complete absence of LPS due to mutations in the genes responsible for lipid A synthesis. The modification of lipid A results from alterations in the *pmrA* and/or *pmrB* genes, with mutations being more prevalent in *pmrB*. It leads to the continuous activation of *pmrA* and enhances the expression of the *pmrCAB* operon. This results in the production and attachment of phosphoethanolamine (PEtN) to the 4′-phosphate or 1′-phosphate group of the lipid A chain [38,46].

LPSs are synthesized within the cytoplasm through the acetyltransferase Lpx pathway and then transported to the OM via the Lpt pathway, a system that moves LPSs across the periplasm for integration into the OM. Mutations, including nucleotide substitutions, deletions, or inactivation by the insertion sequence *ISAba11* in the *lpxA*, *lpxC*, and *lpxD* genes—encoding acetyltransferases critical for lipid A biosynthesis—result in the complete loss of LPSs. Meanwhile, mutations in the *lptD* gene result in the loss of the ability to transport LPSs from the cytosol to the OM [44,46,47]. Thus, the addition of PEtN to lipid A hinders polymyxins from penetrating the OM, while the absence of LPSs significantly diminishes their binding affinity to the OM [48].

Other two resistance mechanisms related to LPS synthesis include decreased expression of cofactors essential for LPS production and decreased levels of proteins responsible for exporting and stabilizing primary OM components. Biotin, which is crucial for fatty acid production, consequently facilitates LPS synthesis. The *lpsB* gene encodes an enzyme called glycosyltransferase, which is crucial for biotin synthesis in *A. baumannii*. Deletions in *lpsB* have been associated with resistance to colistin [44,46]. A linkage between resistance and *A. baumannii* isolates possessing the *vacJ*, *zndP*, and *pldA* genes has been identified. The *VacJ* gene is associated with the Vps–VacJ ABC transporter system responsible for maintaining LPSs in the outer leaflet and phospholipids in the inner leaflet of the OM [44].

Phospholipase D, known as PldA, is responsible for removing phospholipids from the outer leaflet of the OM, whereas a zinc-dependent peptidase ZndP acts prior to PldA and is essential for OM maintenance. Therefore, mutations in these genes contribute to colistin resistance [44,49]. Moreover, colistin resistance in *A. baumannii* is associated with a marked decrease in the production of osmoprotective amino acids, including proline, glycine, and aspartate, which are crucial for maintaining osmotic balance. Changes in the OM of colistin-resistant *A. baumannii* are tipically associated with chromosomally encoded resistance [47].

Resistance to fluoroquinolones in *A. baumannii* primarily arises from spontaneous mutations in the *gyrA* and *gyrB* genes, which encode DNA gyrase, as well as in the *parC* gene, responsible for encoding topoisomerase IV. These mutations alter the structure of the DNA gyrase and topoisomerase IV complexes, preventing fluoroquinolones from binding, disrupting DNA replication, and thereby rendering the bacteria resistant to the drug. Mutations in the *gyrA* gene alone cause moderate resistance, while mutations in both the *gyrA* and *parC* genes lead to high resistance [38,44,50]. Another mechanism of resistance is the protection of DNR gyrase and topoisomerase IV by the proteins from the families of QrnA, QrnS, and QrnS [44].

An essential contributor to high-level resistance of *A. baumannii* to aminoglycosides is the *armA* gene, which encodes 16S rRNA methylase. This enzyme restricts the binding of aminoglycosides to bacterial ribosomes by methylating the ribosomal 30S subunit [38,44].

Ribosomal protection proteins, including TetM, TetW, TetO, and TetS, enable *A. baumannii* resistance to antibiotics by inducing a non-covalent alteration of ribosomes. Minocycline resistance is associated with the ribosomal protection protein encoded by the *tetM* gene. This protein facilitates the displacement of tetracycline from its binding site on the ribosome via a guanosine triphosphate (GTP)-dependent process, allowing protein synthesis to proceed despite the presence of tetracycline [44,50]. Resistance to tigecycline is mediated via the *rpsJ*, *trm*, and *rrf* genes, which subsequently cause an alteration in the drug target [51].

Infections caused by *A. baumannii* show resistance to rifampicin due to mutations in the *rpoB* gene, which encodes the β-subunit of rifamycin-sensitive RNA polymerase. These mutations disrupt RNA elongation shortly after the incorporation of the initial nucleotides. Spontaneous resistance to rifampicin is mainly caused by single-point mutations leading to amino acid substitutions, while it is more rarely linked to specific insertions or deletions [44].

Resistance of *A. baumannii* to macrolides is primarily attributed to the main 23S rRNA (adenine(2058)-N(6))-methyltransferases, encoded by the *erm(B)*, *erm(C)*, and *erm(F)* genes), as well as to the ABC-F-type ribosomal protection protein Msr(E), encoded by the *msr(E)* gene. Cross-resistance to macrolides, lincosamides, and streptogramin B can be explained by shared binding sites on the 23S rRNA [44,50].

Multidrug efflux pumps are the primary mechanisms of insensitivity to oxazolidinones. However, modifications to the target site, such as nucleotide alterations in the V domain of the 23S rRNA, and/or the presence of the transmissible Cfr(B) rRNA 23S methyltransferase, can also contribute to insensitivity. The Cfr-like rRNA 23S methyltransferase is additionally linked to resistance against lincosamides, streptogramins, phenicols, and pleuromutilins. Alterations in the P site of the 50S ribosomal subunit led to a resistance to linezolid, while the *poxtA* gene, encoding the ABC-F ATP-binding cassette ribosomal protection protein, plays a significant role in the development of resistance. The ABC-F ATP-binding cassette ribosomal protection protein is also implicated in A. baumannii resistance to bacterial protein synthesis inhibitors, such as tetracyclines, macrolides, lincosamides, streptogramins, phenicols, and pleuromutilins [44,50].

Trimethoprim insensitivity in *A. baumannii* is primarily mediated by enzymes called trimethoprim-resistant dihydrofolate reductases, including DfrA1, DfrA5, DfrA7, DfrA10, DfrA12, DfrA14, DfrA16, DfrA17, DfrA19, DfrA20, DfrA27, and DfrB1. These reductases are encoded by the corresponding genes *dfrA1*, *dfrA5*, *dfrA7*, *dfrA10*, *dfrA12*, *dfrA14*, *dfrA16*, *dfrA17*, *dfrA19*, *dfrA20*, *dfrA27*, and *dfrB1*. *A. baumannii*’s inherent insensitivity to sulfonamides is linked to sulfonamide-resistant dihydropteroate synthases encoded by the *sul1* and *sul2* genes [44].

Murein transpeptidases, known as penicillin-binding proteins (PBPs), are essential for the synthesis of the bacterial cell wall, as they catalyze the formation of cross-links between peptidoglycan strands. Peptidoglycan is a vital component that maintains the structural stability of the cell wall, protects the cell from osmotic stress, and ensures cell shape. β-lactams inhibit PBPs by mimicking the natural substrate, disrupting the cross-linking process. This interference destabilizes the peptidoglycan layer, leading to cell wall weakness and eventual bacterial lysis during growth [52]. PBPs are classified into two broad categories based on their molecular size: low-sized PBPs (class C PBPs or cPBPs, which include carboxypeptidases and endopeptidases) and high-sized PBPs [53,54]. The high-sized PBPs are further subdivided into class A and class B PBPs. Thus, resistance mediated by PBPs is a major factor contributing to β-lactam resistance in both gram-negative and gram-positive microorganisms. This mechanism involves alterations in the PBPs, which are the primary targets of β-lactam antibiotics [53]. *A. baumannii* contains seven distinct PBPs. These include several class A PBPs (PBP1a and PBP1b), several class B PBPs (PBP2 and PBP3), and three class C PBPs (PBP5/6, PBP6b, and PBP7/8). Additionally, this pathogen has a monofunctional transglycosylase [53]. However, most variations in PBPs were identified as silent mutations, which do not contribute to resistance against β-lactams [55].

Although alterations of PBPs are not an essential mechanism of resistance against β-lactams in *A. baumannii*, they should not be ignored [43,49]. Thus, mutations of the gene encoding PBP2 are related to imipenem resistance, while alterations of the gene encoding PBP3 are related to meropenem, sulbactam, and cefiderocol resistance [44,50]. Some PBPs, including PBP3-H370Y, PBP3-Q488K, and PBP3-Y528H, can be affected by the novel combination of sulbactam and durlobactam [53,56]. This intravenous β-lactam/β-lactamase inhibitor combination was approved by the US FDA in May 2023 for treating adults with hospital-acquired and ventilator-associated bacterial pneumonia caused by susceptible strains of the *Acinetobacter baumannii*–*calcoaceticus* complex, including carbapenem-resistant *A. baumannii* (CRAB) [57].

#### 3.1.3. Enzymatic Drug Inactivation

The production of enzymes that inactivate antibiotics by adding specific chemical moieties or by degrading the drug molecule itself, thus preventing its interaction with the target, is one of the most potent mechanisms bacteria use to evade the effects of antimicrobial agents [58].

The intrinsic insensitivity of *A. baumannii* is closely linked to the inactivation of β-lactams. It has been established that this bacterium presents β-lactamases of Ambler classes A, B, C, and D [50]. The serine β-lactamases of molecular class A are TEM (Temoneira), SCO-1 (a novel plasmid-mediated class A β-lactamase from *E. coli*, exhibiting characteristics similar to carbenicillinase), CARB (carbenicillin-hydrolyzing β-lactamase), SHV (sulfhydryl reagent variable), GES (Guiana extended-spectrum β-lactamase), CTX-M (cefotaxime-hydrolyzing β-lactamase), PER (*Pseudomonas* extended resistant), and KPC (*K. pneumoniae* carbapenemase) [38,59,60,61]. Intrinsically, Ambler class A β-lactamases are narrow-spectrum enzymes responsible for the primary inactivation of antibiotics from the penicillin group and first- and second-generation cephalosporins, such as TEM-1, TEM-2, SCO-1, CARB-4, and SHV [44,59,62,63].

Usually, narrow-spectrum class A β-lactamases can be impeded by the classical β-lactamase inhibitors. However, these enzymes, through point mutations, can develop the capacity to hydrolyze extended-spectrum cephalosporins such as ceftazidime, ceftriaxone, cefotaxime, and aztreonam, the latter being currently the only available monobactam. Thus, due to their ability to destroy potently wide-spectrum β-lactams, these enzymes are named ESBL. Other ESBLs are GES-11, CTX-M, and KPC, including KPC-2, KPC-3, and KPC-5. ESBL encoding genes are widely distributed among gram-negative bacteria by plasmids or mobile genetic elements (MGEs) [64,65].

Among the main genes encoding class A lactamases are *bla_TEM_-92*, *bla_SHV_*, *bla_GES_-11*, *bla_GES_-14*, *bla_PER_-1*, *bla_PER_-7*, and *bla_VEB_-1* [2,44,50,62].

Enzymes known as metallo-β-lactamases (MBLs) possess a Zn^2+^ ion at their active sites and are encoded by mobile genetic elements like plasmids and integrons. These enzymes catalyze the hydrolysis of nearly all β-lactams, including carbapenems, while sparing monobactams, thereby contributing to MDR [44]. MBLs belong to class B and are named as IMP (imipenemase), VIM (Verona integron-encoded MBL), NDM (New Delhi metallo-β-lactamase), and SIM (Seoul imipenemase) [35]. The main genes that encode MBLs are *bla_IMP_*, *bla_VIM_*, *bla_SIM_*, *blaS_PM_*, *bla_GIM_*, *bla_DIM_*, and *bla_NDM_* [64,65].

Chromosomally encoded cephalosporinases, known as *Acinetobacter*-derived cephalosporinases (ADCs), are classified as class C β-lactamases and are present in all *A. baumannii* strains. The gene encoding ADC is *bla_ADC_* (formerly known as *bla_AmpC_*). The insertion of IS*Aba1* or IS*Aba125* sequences upstream of the genes that encode ADCs promotes their overexpression by introducing stronger promoters. ADC enzymes present extended-spectrum resistance to β-lactams, and their covalent modification leads to imipenem resistance [44,66].

Recently, enzymes known as class D β-lactamases, which are capable of inactivating carbapenems and cephalosporins, have been detected in *A. baumannii*. These enzymes are named oxacillinase (OXA)-type carbapenemases. The most predominant oxicillinases in *A. baumannii* include OXA-23-like, OXA-24/40, OXA-48, OXA-51, OXA-58, OXA-143-like, and OXA-235-like. Numerous *bla_OXA_* genes have been identified such as *bla_OXA_-51*, *bla_OXA_-23*, *bla_OXA_-24*, *bla_OXA_-58*, *bla_OXA_-143*, and *bla_OXA_-235*. These genes are located on both chromosomes and plasmids. For instance, acquired carbapenemases include OXA-23, OXA-24, OXA-48, and OXA-58, while OXA-51 is an intrinsic carbapenemase in *A. baumannii*. OXA enzymes, characterized by low catalytic efficiency, along with porin deletion and various other antibiotic resistance mechanisms, can contribute to significantly high insensitivity to carbapenems [2,18,32,38].

Aminoglycoside-modifying enzymes (AMEs) are key factors responsible for bacterial insensitivity to aminoglycosides. Depending on the location of aminoglycoside alteration through *N*-acetylation, *O*-nucleotidylation, and *O*-phosphorylation, AMEs are classified as acetyl-, adenyl-, and phosphor-transferases [2,44].

The acetylation of -NH_2_ groups is catalyzed by aminoglycoside N-acetyltransferases (AACs), which use acetyl coenzyme A as a donor substrate to transfer the acetyl group to the acceptor molecule. Aminoglycoside O-nucleotidyltransferases (ANTs) inhibit the activity of aminoglycosides by catalyzing the transfer of an adenosine monophosphate (AMP) molecule from adenosine triphosphate (ATP) to the -OH group on the antibiotic, with ATP serving as a donor substrate. Changes in the distribution of antibiotic charge and blockage of its interaction with the ribosome occur due to the addition of a phosphate group to the drug by aminoglycoside O-phosphotransferases (APHs). Thus, the drug becomes ineffective due to the action of enzymes that destroy the chemical structure of aminoglycosides, reducing their affinity for the target or impeding ribosomal binding [43,67]. The main families of genes responsible for resistance to aminoglycosides are *aadB*, *apa6*, *aadA*, and *aacc1*. Genes can be transferred by MGEs such as integrons, gene cassettes, transposons, and conjugated elements [38].

The enzymes known as fluoroquinolone-acetylating aminoglycoside 6′-N-acetyltransferase AAC (6′)-Ib-cr and fluoroquinolone-acetylating aminoglycoside 6′-N-acetyltransferase AAC, encoded by *aac(6*′*)-Ib-cr* and *aac(6*′*)-Ib-cr5I*, respectively, cause the inactivation of fluoroquinolones [38].

Rifampin is enzymatically modified and inactivated by rifampin ADP-ribosyltransferases, primarily including enzymes such as Arr, Arr-2, Arr-3, and Arr-4. In contrast, the inactivation of amphenicols involves chloramphenicol O-acetyltransferases, including type A-1, type A-2 CatII, type B-2 CatB11, type B-3 CatB3, and type B-3 CatB8, which are encoded by the genes *catA*1, *catA2*, *catB1*1, *catB3*, and *catB8*, respectively. Another enzyme involved in the inactivation of amphenicols is the bifunctional type B-3 chloramphenicol O-acetyltransferase CatB8/aminoglycoside N-acetyltransferase AAC (6′)-Ib, or catB8/aac(6′)-Ib [44].

Two macrolide 2′-phosphotransferases, Mph(A) and Mph(E), encoded by the mph(A) and mph(E) genes, inactivate macrolides and, when specific regulatory proteins are present, result in resistance to erythromycin, clarithromycin, azithromycin, and oleandomycin [44].

Resistance to fosfomycin is attributed to glutathione transferases from the FosLL, FosA3, and FosA3/FosA4 families, encoded by the *fos*, *fosA3*, and *fosA* genes, respectively. Additionally, fosfomycin resistance is mediated by a bacillithiol transferase from the FosB1/FosB3 family, encoded by the *fosBOne* gene, which contributes to intrinsic *A. baumannii* resistance to fosfomycin. FosA facilitates the attachment of glutathione to the C1 position of fosfomycin, leading to its inactivation, while FosB catalyzes the cleavage of the drug’s epoxide ring using either bacillithiol or L-cysteine [44].

It has been recently found that tetracyclines, including tigecycline, eravacycline, and omadacycline, can be also inactivated by various Tet(X) monooxygenases, encoded by plasmid-mediated *tet(X3)*, *tet(X4)*, and *tet(X5)* genes [44,50,51].

#### 3.1.4. Drug Efflux

The active efflux of antimicrobial medicines from bacterial compartments, along with diffusion across the membrane, serves as an important molecular and biochemical mechanism of both intrinsic and acquired MDR. It also interacts with various mechanisms, including the destruction or alteration of drugs and the modification or preservation of antibiotic targets, significantly elevating resistance levels and profiles [68].

Efflux pumps located in the IM or OM of bacteria are usually a complex of bacterial machineries that can expel toxic compounds out of the bacterial cell [43]. A critical characteristic of these complexes is their ubiquity across nearly all bacterial species, contributing to antibiotic resistance through their common underlying resistance mechanisms [43]. These efflux pumps have been classified into five major superfamilies: the ATP-binding cassette (ABC) superfamily, the RND family, the MFS, the multidrug and toxic compound extrusion (MATE) family, and the small multidrug resistance (SMR) family [43,50]. Recently, a new superfamily referred to as the proteobacterial antimicrobial compound efflux (PACE) was identified [43].

Efflux pumps are categorized based on their energy source into two groups: primary efflux pumps, which utilize ATP (e.g., the ABC superfamily), and secondary efflux pumps, which rely on the proton motive force (PMF) [43,50].

Most efflux pumps in gram-negative pathogens are part of the RND superfamily and typically possess a tripartite structure consisting of an outward conduit or outer membrane protein in the OM and subsequently a membrane fusion protein (MFP) and an active pump or inner membrane protein in the IM [43,69]. The RND family is a class of secondary efflux pumps that are primarily arranged in an operon structure. These pumps play a significant role in AMR by enhancing insensitivity to multiple antibacterial drugs, including tetracyclines, fluoroquinolones, aminoglycosides, and the penicillin group [43].

In *A. baumannii*, insensitivity to β-lactams, particularly carbapenems and cephalosporins, is associated with the overexpression of the AdeABC efflux pump, which belongs to the RND superfamily. It consists of three components: AdeB, which expels antibiotics from the cell; AdeA, which functions as a membrane fusion protein; and AdeC, which serves as an outer membrane protein. AdeB can transport a wide range of substrates, from hydrophilic to hydrophobic, with either positive charges or neutral properties. The expression of this efflux pump is controlled by the AdeRS two-component regulatory system (TCRS) [38,44].

TCRSs enable bacteria to respond to environmental changes [70]. Point mutations within the *adeRS* operon can upregulate pump expression, thereby increasing antibiotic resistance. Recently detected RND AdeIJK and MATE AbeM efflux pumps may also play a role in the emergence of resistance to imipenem and cephalosporin [38,44].

The MATE AbeM efflux pump also removes fluoroquinolones, chloramphenicol, erythromycin, tetracyclines, and aminoglycosides [71]. *A. baumannii* is able to expel aminoglycosides through various drug efflux mechanisms, including the MDR efflux MFS transporter AmvA; the MDR efflux RND transporter AdeABC, which comprises the OM channel subunit AdeC, the periplasmic adaptor subunit AdeD, and the permease subunit AdeE; and the MDR efflux SMR superfamily transporter EmrE. Additionally, the expression of these transporters is regulated by the DNA-binding response regulator transcription factor AdeR and the two-component sensor histidine kinase AdeS. These components are encoded by the *amvA*, *adeC*, *adeD*, *adeE*, *adeR*, *adeS*, and *emrE* genes [44].

It has been established that another SMR AbeS transporter can extrude β-lactams, chloramphenicol, ciprofloxacin, and erythromycin. However, it has a limited impact on reducing the susceptibility of *A. baumannii* to these compounds [71].

Efflux pumps from the RND superfamily, encoded by the *adeA*, *adeB*, and *adeC* genes, along with the tetracycline MFS transporter, where TetA and TetB (encoded by *tetA* and *tetB*) play key roles, contribute to tetracycline resistance. The *tetA* gene may be contained in a transposon while *tetB* is carried by a plasmid. RND efflux pumps AdeABC, Ade-FGH:RND, and AdeIJK function synergistically with TetA. For example, TetA expels tigecycline into the periplasm, allowing the RND pumps to subsequently transport the drug across the OM. Other MFS pumps are TetC, TetD, TetG, and TetH [44].

The predominant RND efflux pump responsible for the effective elimination of tetracyclines is AdeABC. The AdeABC pump is controlled by a TCRS called AdeRS. Point mutations in the *adeRS* operon can upregulate the pump’s expression, resulting in increased antibiotic resistance [44].

The integration of a sequence known as IS*Aba1* into the *adeS* gene leads to the overexpression of AdeABC. Although the RND efflux pump AdeIJK contributes less significantly to tetracycline resistance in *A. baumannii*, it can cooperate with other overexpressed pumps, such as AdeABC and AcrAB-TolC, to enhance tigecycline resistance [44,50]. Additionally, AdeIJK and AcrAB-TolC have been identified as potential mechanisms contributing to rifamycin resistance in *A. baumannii* strains [44].

Efflux pumps such as AdeABC, AdeDE, AdeFGH, and AdeIJK contribute to resistance against aminoglycosides, tetracyclines, fluoroquinolones, macrolides, lincosamides, and chloramphenicol. However, among them, AdeABC is the most predominant drug expulsion system in this bacterium [2,27,32].

LmrS, an MFS pump, is able to extrude trimethoprim as well as aminoglycosides, macrolides, oxazolidinones, and amphenicols. Resistance of *A. baumannii* to trimethoprim is associated with the RND efflux pump MexAB-OprM, which consists of MexA, a membrane fusion protein; MexB, a carrier in the IM; and OprM, a protein embedded in the OM. This transporter participates in the resistance of many antibiotics, including sulfonamides [66].

Another efflux pump that eliminates trimethoprim from the bacterial cell is the aforementioned AdeIJK. The RND plasmid-borne OqxAB transporter has been linked to resistance against trimethoprim, quinolones, tetracyclines, glycylcyclines, and nitrofurans, while the MFS AbaF efflux pump, encoded by the *abaF* gene, expels fosfomycin [44,50]. EmrAB-like efflux pumps in *A. baumannii* reduce its sensitivity to polymyxins due to an increased expression of *emr*-*B* like genes [47]. The upregulation of genes *adeI*, *adeC*, *emrB, mexB*, and *macAB*, which encode the protein components of efflux pumps, has been observed in colistin-resistant strains of *A. baumannii*. A recent study has linked an amino acid substitution (N104M) in the gene *ttg2C*, which encodes the toluene tolerance efflux pump Ttg2C, with high-level colistin resistance [66].

Thus, in this bacterium, the RND superfamily efflux pumps are related to insensitivity to aminoglycosides, β-lactams, macrolides, lincosamides, polymyxins, chloramphenicol, tetracyclines, fluoroquinolones, rifamycins, and folic acid inhibitors, while the MFS superfamily, to tetracyclines, macrolides, oxazolidinones, chloramphenicol, aminoglycosides, and fosfomycin; the MATE superfamily, to β-lactams, fluoroquinolones, chloramphenicol, erythromycin, tetracyclines, and aminoglycosides; and finally the SMR superfamily, to fluoroquinolones, aminoglycosides, chloramphenicol, erythromycin, and tetracyclines [44,71].

### 3.2. P. aeruginosa

The intrinsic and acquired resistance pathways enable *P. aeruginosa* to overcome the effects of antimicrobials [2,43]. However, the primary mechanisms of resistance include the overexpression of efflux pumps, reduction in OM integrity, and the acquisition or mutation of resistance genes that encode proteins involved in controlling the passive diffusion of antibiotics through the OM [2].

#### 3.2.1. Drug Uptake Limitation

It has been established that *P. aeruginosa* lacks major porins, including OmpF and OmpC, leading to an OM resistance to antibiotic penetration that is 100-fold lower compared to *E. coli*. Moreover, a vast family of OprD (OccD1) porins, consisting of 19 representatives, has also been associated with a decreased permeability in this bacterium [72,73].

These porins are unique channel proteins that possess specific binding sites for a particular set of molecules, i.e., participate in the absorption of various nourishing substances, but mainly amino acids. Reduced permeability caused by the downregulation of OprD proteins is the key pathway of intrinsic resistance to carbapenems, including imipenem and meropenem [72,73]. Thus, carbapenem resistance has been associated with alterations that result in the downregulation of *oprD* manifestation, as well as with amino acid substitution mutations in OprD found in hypermutator isolates [73].

It has been also established that epigenetic regulation of *opdQ* expression causes an elevation in the MIC of meropenem. This gene encodes the OpdQ porin, which is a member of the OprD family [73]. In addition, a large quantity of OprD proteins which lead to the carbapenem resistance have been found in biofilms and OMV [74].

Another protein found in the OM of *P. aeruginosa* is OprH, the smallest porin among all porins in this pathogen, belonging to the class of gated porins. OprH facilitates the absorption of bivalent cations, engages with LPSs via electrostatic bonds, and subsequently strengthens the OM. Due to it, OprH contributes to the antibiotic resistance, especially to polymyxins and aminoglycosides, which penetrate the bacterial cell membrane through interaction with LPSs. Overexpression of OprH due to Mg^2+^ starvation has been linked to increased insensitivity to polymyxin B and gentamicin, which is attributed to enhanced activity of the PhoP/PhoQ system [72,73,75].

The PhoP-PhoQ pathway is a TCRS that plays a role in polymyxin resistance by controlling the *arnBCADTEF-pmrE* operon, which encodes LPS alteration enzymes [76]. Non-specific porin OprF is responsible for preserving the OM structure. Alterations in OprF showed that its N-terminal is essential for protein synthesis and membrane integration, whereas the C-terminal is crucial for stable interaction with peptidoglycan and anchoring on the OM. It has been established that *P. aeruginosa* virulence and its protection against macrophages are related to OprF [73]. Overexpression of OprF has been found in the formation of *P. aeruginosa* biofilm and OMV, which consequently prevent antibiotic entry [43,74]. However, some investigations have suggested the total absence of OprF alone does not seem to be a significant pathway of antibiotic insensitivity in clinical strains [74]. Finally, other proteins such as the particular porins OprB, OprE, OprO, and OprP as well as the gated porin OprC are involved in metabolic and ion homeostasis, whereas proteins like OprM, OprN, and OprJ are classified as efflux porins [68].

#### 3.2.2. Drug Target Modification

*P. aeruginosa* develops resistance to aminoglycosides with the help of RNA methylases encoded by the *rmtA* and *rmtB* genes, which are located on transposons and plasmids, as these enzymes methylate the 16S ribosomal RNA (rRNA). The 16S rRNA methylases methylate, a specific nucleotide in the A-site of 16S rRNA, thereby protecting the ribosome from the action of these drugs. Resistance to fluoroquinolones occurs through changes in topoisomerase IV and DNA gyrase due to target site alteration. For example, insensitivity to ciprofloxacin most commonly arises from alterations in the subunits GyrA and GyrB of DNA gyrase or the subunits ParC and ParD of topoisomerase. Thus, resistance to DNA synthesis inhibitors is linked to alterations in the motifs of the *gyrA* and *gyrB* genes within the quinolone-resistant determining region (QRDR motif), which forms part of the active site of the DNA gyrase enzyme [43,74].

Alterations in the amino acid sequences encoding the specific QRDR motif lead to modifications in the A and B subunits of the enzyme, resulting in a reduced binding affinity for fluoroquinolone. The alterations in topoisomerase IV have been identified as point mutations in the amino acid sequences encoded by the *parC* and *parE* genes, which form the ParC and ParE enzymatic subunits, leading to resistance to these antibacterial drugs. *P. aeruginosa* is considered highly resistant when it carries mutations in both the *gyrA* and *parC* genes or simultaneously harbors mutations in the *nalB*, *nfxB*, and *nfxC* genes, leading to their overexpression and, consequently, the overactivation of the MexAB-OprM, MexCD-OprJ, and MexEF-OprN efflux pumps. However, low insensitivity to fluoroquinolones, such as ciprofloxacin, has been observed in this pathogen when mutations in *gyrB*, *parC*, or *parD*, or the presence of efflux pumps, are the only factors involved [43,74].

This pathogen’s insensitivity to β-lactams also relies on alterations in PBPs, primarily in the enzyme PBP3, or in the overexpression of genes encoding these proteins. As a result, β-lactams are unable to bind to their site of action [43]. Alterations in PBP3 lead to insensitivity to ceftazidime, ceftazidime-avibactam, cefepime, piperacillin-tazobactam, ceftolozane-tazobactam, and meropenem. Structural and functional studies have shown that PBP5 in *P. aeruginosa* exhibits β-lactamase activities, thus contributing to β-lactam insensitivity. Moreover, mutations in the nonessential *dacB* gene, encoding PBP4, are linked to the overexpression of AmpC. This leads to an increased resistance to β-lactam antibiotics, specifically lowering susceptibility to antibacterials such as piperacillin, cefotaxime, ceftazidime, cefepime, and aztreonam [77].

Polymyxin-induced resistance in *P. aeruginosa* results from changes in the bacterial LPSs. The electronegativity of LPSs is reduced due to modifications in the lipid A region through the insertion of 4-amino-4-deoxy-L-arabinose (L-Ara4N) and PEtN, which are upregulated by the respective operons *arnBCADTEF* and *pmrCAB*. The mentioned operons are regulated via TCRSs, such as PmrA/PmrB, PhoP/PhoQ, ParR/ParS, CprR/CprS, and ColR/ColS. However, in *P. aeruginosa* the primary pathway for polymyxin insensitivity is the function of the arnBCADTEF operon, responsible for the insertion of L-Ara4N into lipid A [43]. This occurs when L-Ara4N is added to the phosphate groups within lipid A and the main carbohydrate components of the LPSs [73]. As it has been aforementioned, the overproduction of the OMP OprH contributes to polymyxin resistance, as OprH interacts with divalent cation sites on LPSs, hindering the binding of polymyxin [43].

There are eight distinct PBPs in *P. aeruginosa*: class A PBPs, including PBP1a and PBP1b; class B PBPs, consisting of PBP2, PBP3, and PBP3x; and class C PBPs, such as PBP4, PBP5, and PBP7 [53,78]. Among these, PBP3, encoded by the *ftsl* gene is crucial for the growth of this pathogen [53,78,79,80]. Mutations in the *ftsI* gene lead to alterations in the PBP3 sequence, which are closely linked to insensitivity to β-lactams, especially cephalosporins and carbapenems, due to a reduced binding affinity for these drugs [79,81,82]. However, PBP1 and PBP3 in *P. aeruginosa* are still sensitive to the combination of ceftolozane and tazobactam [53,83,84].

Mutations in the enzyme PBP4 have been found to trigger the overexpression of AmpC β-lactamase, thereby contributing to resistance, primarily to penicillins and cephalosporins [53,78].

#### 3.2.3. Enzymatic Drug Inactivation

As with *A. baumannii*, *P. aeruginosa* also generate various β-lactamases, which are classified according to Ambler classes A-D. The most common β-lactamases in *P. aeruginosa* are penicillinases from the molecular class A serine β-lactamases, including *Pseudomonas*-specific enzymes (PSE), CARB, and TEM, with PSE being the predominant one [85]. Chromosomally encoded Ambler class C β-lactamase AmpC plays a crucial role in the natural resistance of *P. aeruginosa* [74,85].

Although AmpC β-lactamase is naturally synthesized at low levels in *P. aeruginosa*, its overproduction can occur either due to induction of the *ampC* gene or through derepression, leading to constitutive high-level expression of the enzyme. Thus, chromosomal *bla_AmpC_* gene activity is promoted by aminopenicillins and cephalosporins, leading to the immediate inactivation of β-lactams via hydrolysis by AmpC. AmpC activation is triggered by two distinct processes, impairment of the peptidoglycan recycling pathway and the depletion of PBP4. Normally, during peptidoglycan synthesis, the release of anhydromuropeptides (anhMPs) occurs [43,85].

Next, anhMPs are transported across the IM into the cytoplasm and are subsequently taken up for re-use in the peptidoglycan biosynthesis process. When anhMP processing is disrupted, these molecules accumulate in the cytoplasm, interact with AmpR, a key regulator of AmpC, and act as signaling molecules, triggering the activation of β-lactamase production in this bacterium [43,85].

Another mechanism associated with AmpC β-lactamase activity involves the inactivation of the *dacB* gene, which encodes PBP4. This inactivation results in the overexpression of the *ampC* gene and the activation of the CreBC (BlrAB) TCRSs. PBP4 plays an important role in producing an anhMP that regulates AmpR. When PBP4 is lost, the anhMP is excluded from the peptidoglycan recycling process, modifying AmpR activity and leading to the activation of *ampC* expression [74].

Consequently, antimicrobial insensitivity to β-lactam antibiotics, including drugs from the penicillin group, cephalosporins (such as cefepime and ceftazidime), tazobactam, avibactam, and aztreonam, emerges. AmpC β-lactamase has minimal effect on carbapenem susceptibility, unless other resistance mechanisms, such as efflux pump overproduction, decreased OprD, and/or the production of class A/class B carbapenemases, are involved [43,85].

Transferable class C β-lactamases, disseminated through horizontal gene transfer, are infrequent in microorganisms such as *P. aeruginosa*, which already possess chromosomally encoded *ampC*. Nonetheless, these transferable β-lactamases can still target penicillins, cephalosporins, and monobactams. It is believed that these enzymes originally derived from chromosomally encoded versions, which were later transferred to MGEs [79]. Mutations in ampC can reduce the binding affinity of β-lactamase inhibitors like avibactam, tazobactam, and relebactam to AmpC [79].

Class B enzymes, specifically MBLs produced by *P. aeruginosa*, catalyze the hydrolysis of β-lactam rings through pathways that require a zinc ion. High insensitivity of *P. aeruginosa* to carbapenems has been observed in the presence of MBLs, as these enzymes can degrade carbapenems and are critical in managing diseases caused by this pathogen [79]. The most prevalent MBLs are carbapenemases VIM and IMP that are horizontally acquired. It has been identified that these two enzymes have multiple variants, while other three enzymes—SPM (Sao Paulo MBL), NDM, and GIM—present only one variant each, i.e., SPM-1, NDM-1, and GIM-1, respectively [43,86]. The genes encoding VIM and IMP are often found on integrons, which enables these genes to integrate into either the chromosome or plasmids, facilitating their spread throughout bacterial populations [74]. β-lactamase inhibitors impede the activity of MBLs [77].

As mentioned earlier, ESBLs are a type of β-lactamases that inactivate β-lactams, especially oxyimino-β-lactams and monobactams, and are susceptible to inhibition by β-lactamase inhibitors such as clavulanic acid. They are encoded by plasmids and can easily be transmitted from one organism to another. The most prevalent ESBLs in *P. aeruginosa* are class A β-lactamases—TEM, CTX-M, and SHV—encoded by genes *bla*_TEM_, *bla*_CTX-M_, and *bla*_SHV_, respectively [87]. Other Ambler class A ESBLs found in *P. aeruginosa* are VEB (Vietnam extended β-lactamase), PER, GES, PSE, and BEL (Belgium extended β-lactamase)-type enzymes [43,74].

Ambler class A ESBLs degrade oxyimino-aminothiazolyl cephalosporins (such as cefotaxime, cefuroxime, cefepime, ceftriaxone, and ceftazidime), along with other cephalosporins like cefpirome, as well as penicillins and aztreonam. However, they do not affect cephamycins (e.g., cefoxitin) or carbapenems [43,74]. TEM, SHV, CTX-M, BEL, PER, and VEB can be inhibited by clavulanic acid, sulbactam, tazobactam, avibactam, vaborbactam, and relebactam. Contrarily, the members of the PER family—PER-1 and PER-2—are the predominant variants and exhibit reduced susceptibility to inhibition by clavulanic acid, tazobactam, and avibactam [77].

A newly identified chromosomally encoded Ambler class A β-lactamase, PIB-1 (*Pseudomonas* imipenem β-lactamase), which degrades imipenem, has been discovered. However, PIB-1 is not able to inactivate other β-lactams, including cephalosporins [88,89]. Another novel β-lactamase, the German *Pseudomonas* carbapenemase (GPC-1), has recently been detected. Strains of *P. aeruginosa* strains that produce GPC-1 may demonstrate weak carbapenemase activity, indicating that they do not effectively hydrolyze carbapenems to a significant degree. Consequently, these strains may go undetected by standard diagnostic tests [90].

Moreover, *P. aeruginosa* can produce Ambler D class β-lactamases such as OXA-type β-lactamases. The majority of genes encoding these OXA-type β-lactamases are integrated into the bacterial genome via horizontal gene transfer, except for the native OXA-type enzyme OXA-50, which is capable of destroying carbapenems [43,79]. The OXA-type β-lactamases commonly identified in this pathogen include OXA-51, OXA-2, and OXA-10. These enzymes cause insensitivity to ureidopenicillins and carboxypenicillins. It has also been found that OXA-10-like β-lactamases can degrade ceftazidime, a third-generation cephalosporin [43,79].

The OXA-2 and OXA-10 β-lactamases, which arise from point mutations, are commonly found on integrons and plasmids. These MGEs promote the transfer of genes encoding these enzymes across various gram-negative bacteria [43]. Both of these β-lactamases exhibit increased hydrolysis of cephalosporins such as cefepime and ceftazidime, as well as aztreonam, resulting in insensitivity to most β-lactamase inhibitors [43]. Contrary to *A. baumannii*, not all OXA enzymes produced by *P. aeruginosa* are able to inactivate carbapenems. Enzymes like OXA-23, OXA-40, and OXA-58, which can degrade carbapenems, have been identified [79,91].

Carbapenemases of the Ambler class A KPC group have also been identified in *P. aeruginosa*. The genes encoding KPC enzymes are acquired through horizontal gene transfer from *Enterobacteriaceae* spp. [43]. The production of AMEs such as APHs, AACs, and ANTs are among the resistance mechanisms to aminoglycosides in *P. aeruginosa* [92]. However, the most prevalent enzymes found in this pathogen are from the ANT and AAC classes [43].

Thus, *P. aeruginosa* APHs transfer a phosphoryl group to the 3′-hydroxyl of aminoglycosides such as kanamycin, neomycin, and streptomycin, effectively inactivating these antibiotics. Meanwhile, *P. aeruginosa* AACs transfer an acetyl group to the amino group at positions 3′ and 6′ of aminoglycosides, leading to the inactivation of gentamicin, tobramycin, netilmicin, kanamycin, and amikacin. Resistance to gentamicin, amikacin, and tobramycin in *P. aeruginosa* is mediated by ANTs, which transfer an adenylyl group to either the amino or hydroxyl group of these aminoglycosides [72]. The genes responsible for encoding AMEs are prevalent in *P. aeruginosa* isolates, with the primary genes being *aac(6*′*)-I*, *aac(6*′*)-II*, *ant(2*″*)-*I, and ap*h(3*′*)-VI* [93].

#### 3.2.4. Drug Efflux

The principal efflux system in *P. aeruginosa* facilitating the removal of antibacterial agents is the RND superfamily of pumps, characterized by a tripartite structure. This pathogen contains at least 12 RND efflux pumps, with four, including MexAB-OprM, MexCD-OprJ, MexEF-OprN, and MexXY-OprM, serving pivotal roles in mediating antibacterial insensitivity via efflux mechanisms [43,74].

Efflux systems and the low-permeability OM collaborate to enhance antimicrobial resistance in bacteria; therefore, antibacterial drugs that successfully cross the OM are often rapidly expelled into the environment by efflux pumps. These pumps not only aid in removing antimicrobials but also play a role in bacterial stress responses. Stress signals, including host factors, detergents, and endogenous bacterial inducers, can promote the selection of efflux pump-expressing, antimicrobial-resistant mutants, even without direct exposure to antimicrobials [74].

Thus, in *P. aeruginosa*, the efflux pumps of the RND superfamily are composed of a periplasmic membrane fusion protein (e.g., MexC, MexA, MexX, and MexE), an RND transporter unit (such as MexD, MexB, MexY, and MexF), and an OM factor (including OprJ, OprM, and OprN). The most abundant contributor to antimicrobial resistance is the MexAB-OprM efflux pump [43]. This system is constitutively operational in natural strains of *P. aeruginosa* and typically does not result in high resistance to antimicrobial agents. However, its upregulation plays a significant role in enhancing *P. aeruginosa* resistance to various antibiotics, including macrolides, fluoroquinolones, a wide range of β-lactams (e.g., penicillins and carbapenems, with the exception of imipenem and biapenem), tetracyclines, chloramphenicol, lincomycin, novobiocin, as well as the antiseptic triclosan and the surfactant sodium dodecyl sulfate [43,74].

The proteins of the MexAB-OprM efflux pump are encoded by the *mexAB-oprM* operon, whose expression is controlled by the transcriptional regulator protein MexR. Thus, MexR acts as a local transcriptional repressor, suppressing the expression of both the *mexAB-oprM* operon and its own gene. Mutations in the *mexR* gene, along with premature truncation of its encoded peptide, are commonly observed in resistant strains of this bacterium. These mutations lead to an elevated expression level of the MexAB-OprM efflux pump [75]. Other repressor genes that regulate the *mexAB-oprM* operon are *nalC* and *nalD*. Single-nucleotide polymorphisms in these genes can also contribute to antimicrobial resistance by regulating the activity of this pump [43,94]. Increased expression of the MexAB-OprM efflux pump has been observed in *P. aeruginosa* strains resistant to carbapenems, as they also produce carbapenemases. The overexpression of the gene encoding the MexAB-OprM pump, coupled with AmpC activity, has been demonstrated to enhance antibiotic resistance synergistically; however, this drug expulsion system is not able to extrude imipenem, imipenem/relebactam, ceftolozane/tazobactam, or colistin [43].

The MexXY-OprM system contributes to the natural insensitivity of the pathogen to aminoglycosides, tetracycline, erythromycin, and cefepime [43]. The RND family pumps and the operon *mexXY*, which encodes the subunits of the MexXY-OprM efflux system, lack the gene for the OM channel. However, it has been discovered that MexXY can form a fully functional RND efflux pump when coupled to OprM, which typically forms part of the MexAB-OprM efflux system [74,75].

Interestingly, a distinct gene named *oprA*, positioned downstream of the *mexY* gene, has been identified as part of an operon with *mexXY* in certain strains of *P. aeruginosa*, encoding the OM channel OprA. OprA exhibits 47% sequence similarity to OprM; therefore, specific antibiotics, like carbenicillin and sulbenicillin, have been recognized as targets of the MexXY-OprA efflux system [69]. The MexXY-OprM efflux pump is controlled by the MexZ repressor protein, and mutations within or near the mexZ gene cause a loss of repression, leading to an overproduction of the efflux pump and increased expression [95].

Additionally, MexXY has been found to correlate with aminoglycoside-associated resistance as it works synergistically with AMEs. Overexpression of *mexXY* has been particularly noted in strains that produce these enzymes. Moreover, it has been established that MexXY plays a role in resistance to various antipseudomonal drugs, similar to the MexAB-OprM efflux system [43].

Other efflux systems, though not prominent, detected in *P. aeruginosa* strains include the MexCD-OprJ and MexEF-OprN pumps. However, their expression correlates with antimicrobial resistance [43]. The MexCD-OprJ efflux pump has been identified as a key factor in the resistance to fluoroquinolones, tetracyclines, chloramphenicol, as well as various detergents, dyes, and organic solvents. In natural-type strains of *P. aeruginosa*, this efflux system remains inactive under normal conditions [95]. However, exposure to agents like benzalkonium chloride, chlorhexidine gluconate, tetraphenylphosphonium chloride, ethidium bromide, rhodamine 6G, and acriflavine can induce its activity. The activity of the *mexCD-oprJ* operon is controlled by the local repressors NfxB and EsrC. Mutations in the *nfxB* gene are commonly observed in clinical strains of *P. aeruginosa*, driving overproduction of the MexCD-OprJ efflux pump and leading to high levels of insensitivity to multiple antibacterial drugs. The regulation of the *nfxB* promoter depends on the transcription factor VqsM. The absence of VqsM suppresses *nfxB* expression and enhances *P. aeruginosa* insensitivity to a wide range of antibacterials [69].

The EsrC protein functions as a second local repressor by binding to the promoter region of the *mexCD-oprJ* operon and modulating its expression in coordination with NfxB. Additionally, NfxB negatively regulates the transcription of the *esrC* gene [69]. As a result, the NfxB protein acts as a negative regulator of the MexCD-OprJ efflux pump. Mutations within the nfxB gene lead to different levels of MexCD-OprJ expression, which contributes to varying degrees of resistance to antibacterial agents in clinical isolates [95].

The MexEF-OprN system expels various antibacterial drugs, including trimethoprim, chloramphenicol, tetracycline, and imipenem, from the bacterial cell. In contrast to the local repressors regulating the MexAB-OprM and MexCD-OprJ efflux systems, the expression of the MexEF-OprN efflux pump is controlled by a LysR-like activator MexT. Encoded by the *mexT* gene, located upstream of the *mexEF-oprN* operon, MexT promotes the expression of *mexEF-oprN* by binding to the *nod* box in the promoter region. Similar to the MexCD-OprJ system, the MexEF-OprN pump remains inactive under normal conditions. However, mutations in MexT can activate this regulator, leading to the overproduction of the MexEF-OprN efflux pump and repression of the OprD porin. This results in the formation of nfxC-type MDR *P. aeruginosa* mutants [75,96].

A mutated *mexS* gene has been identified in these strains, which regulates the *mexEF-oprN* operon. The MexS protein functions as a repressor of the *mexT* gene, thus limiting the expression of the *mexEF-oprN* operon. Disruption of *mexS* results in an accumulation of electrophilic metabolites, which activate MexT, leading to the increased expression of *mexEF-oprN* [75,96].

Other proteins that activate the expression of the *mexEF-oprN* operon include BrlR and the transcriptional regulator CmrA. BrlR activates the expression by binding directly to the *mexEF-oprN* promoter, while CmrA enhances the expression of *mexT* and *mexEF-oprN* via its regulation of MexS and PA2048, a protein regulated by CmrA [75,96].

### 3.3. K. pneumoniae

*K. pneumoniae* naturally exhibits insensitivity to penicillin antibiotics, contributing to its role as a major global health issue. The emergence of MDR strains, which produce beta-lactamases such as ESBLs and carbapenemases, further complicates treatment options. Additionally, *K. pneumoniae* rapidly acquires genetic elements, leading to the emergence of two distinct pathotypes: classical *K. pneumoniae* (cKp) and hypervirulent *K. pneumoniae* (hvKp). The hypervirulent strain, more commonly associated with community-acquired infections, raises particular concern due to its increased virulence. The global spread of both the MDR *K. pneumoniae* and hvKp strains remains a pressing public health challenge [43,97].

#### 3.3.1. Drug Uptake Limitation

OM porins, including OmpK35, OmpK36, OmpK37, OmpK38, OmpK26, and PhoE, are present in *K. pneumoniae*. Among these, OmpK35 (also known as OmpF) and OmpK36 (a member of the OmpC group) are classified as the major porins responsible for the uptake of antibiotics, such as β-lactams and fluoroquinolones, into the cell [43,98].

Although OmpK35 is not considered the primary pathway for *K. pneumoniae* resistance, studies have shown that tigecycline-resistant *K. pneumoniae* isolates exhibit reduced OmpK35 expression. This reduction is linked to the *K. pneumoniae* resistance to third-generation cephalosporins and carbapenems [43,99]. In certain instances, OmpK35 may be substituted by OmpK36, which features a smaller channel diameter. Strains lacking OmpK35 and expressing only OmpK36 typically display intermediate carbapenem susceptibility. In contrast, strains with no expression of either OmpK35 or OmpK36 are resistant to both carbapenems and cephalosporins, due to the absence of genes encoding these porins [27].

Mutations in OmpK36, combined with carbapenemase production, have been observed in multiple multi-drug resistant (MDR) *K. pneumoniae* strains. A key mutation involves the insertion of a glycine-aspartate di-amino sequence in extracellular loop three of OmpK36, which reduces the pore size and limits carbapenem entry. This mechanism, mediated by OmpK36, has been frequently detected in the highly virulent ST258/512 strain, which has spread across South and North America, as well as Europe [43]. Additionally, the loss of OmpK36, along with the production of ESBLs and/or AmpC, contributes to insensitivity to carbapenems in this bacterium [100].

The regulation of porin expression can occur through mutations in either the coding regions or promoter sites, as well as through insertional deletions, which may lead to altered or absent porins. For instance, OmpK36 deficiency has been linked to the occurrence of premature stop codons in the *ompK36* gene [99,100]. Furthermore, the reduced ability of the membrane to allow antibiotic passage in *K. pneumoniae* contributes to resistance against multiple drug classes, including β-lactams, fluoroquinolones, chloramphenicol, colistin, aminoglycosides, nalidixic acid, and tetracyclines [99].

#### 3.3.2. Drug Target Modification

Resistance mechanisms involve modifications to LPSs, specifically by altering the phosphate moieties of lipid A with sugars or ethanolamine. Colistin resistance in *K. pneumoniae* is primarily attributed to genetic alterations in transcriptional regulatory systems that control LPS modifications. Key regulators include TCRs PhoPQ and PmrAB, which influence the expression of genes responsible for modifications such as PEtN addition (e.g., *pmrC*) and 4-amino-4-deoxy-l-arabinose (Ara4N) biosynthesis and transfer (e.g., *pbgP*, *pmrE*, and *pmrHFIJKLM*). Proteins such as PmrD and MgrB play pivotal roles in linking the PmrAB and PhoPQ pathways, with MgrB acting as a negative regulator of PhoPQ signaling. Additionally, the CrrAB system modulates PmrAB and PhoPQ expression. Alterations in genes such as *pmrAB*, *phoPQ*, *mgrB*, and *crrB* have been identified as contributors to colistin resistance. An alternative LPS modification pathway includes interactions between TupA-like glycosyltransferases and the CrrAB system [100,101,102].

Alterations in the *lpxM* and *ramA* genes disrupt lipid A maturation and neutralization, while changes in the *pbgP* and *pmrE* genes modify the addition of aminoarabinose. Modifications in the *pmrC* gene lead to an altered incorporation of PEtN, and variations in *pagP* impact the addition of palmitate. Additionally, mutations in regulatory genes such as *phoPQ*, *pmrA*, *pmrD*, and *mgrB* influence LPS modification, while alterations in the *mcr*-*1* gene affect phosphoethanolamine transferases involved in binding PEtN [97,102].

Fluoroquinolone resistance in *K. pneumoniae* is associated with point mutations in the chromosomal genes *gyrA*, *gyrB*, *parC*, and *parE*, which encode the subunits of DNA gyrase and topoisomerase IV. These mutations generally arise spontaneously in the *gyrA* and *parC* genes and lead to alterations in the 5′ quinolone-binding regions of the resulting enzymes. Additional mutations in subunit B contribute to higher insensitivity levels by affecting both enzymes, resulting in the highest fluoroquinolone resistance [43].

Another class of quinolone insensitivity genes includes plasmid-mediated quinolone resistance (PMQR) determinants, which play a critical role in fluoroquinolone resistance. These genes encode proteins such as QnrS, QnrA, and QnrB, which bind to DNA gyrase, protecting it from the effects of fluoroquinolones and thereby contributing to resistance [43,97].

RNA methylases, including ArmA, RmfH, RmfA, and NmpA, responsible for 16S rRNA methylation, have been detected in *K. pneumoniae*. Alterations to the aminoglycoside binding site contribute significantly to antimicrobial resistance in this pathogen. Genes encoding 16S rRNA methylases, such as rmtB, rmtC, and nmpA, are often disseminated between organisms through plasmids, which frequently carry additional resistance factors, such as ESBLs, MBLs, and carbapenemases, promoting the emergence of MDR strains. For example, the uncommon and threatening coexistence of *rmtB*, *armA*, *bla_KPC-2_*, and *iuc* virulence operon-encoding plasmids in *K. pneumoniae* has been recently established. However, the chromosomal localization of these genes has not been determined yet [43,97,99].

Resistance to tetracyclines, particularly tigecycline, can arise due to point mutations in the *rpsJ* gene, responsible for ribosomal protein S10, a crucial part of the 30S ribosomal subunit. Alterations in this ribosomal protein may underlie this phenomenon. Similarly, modifications in ribosomal protein S3 can also contribute to insensitivity. Notably, mutations in the *rpsJ* gene can lead to tigecycline resistance independently of efflux pump involvement. Another pathway contributing to tigecycline insensitivity involves genes encoding 16S rRNA methylases [103].

There are few studies that thoroughly investigate the impact of alterations in PBPs on the resistance to beta-lactam antibiotics in *K. pneumoniae* [104]. However, recent evidence has linked PBPs to the growth and resistance of *K. pneumoniae* to β-lactam antibiotics. Specific enzymes known as zinc finger nucleases have been shown to significantly reduce β-lactam resistance in this pathogen. Furthermore, the knockdown of PBPs has been found to counteract the inhibitory impacts of zinc finger nucleases on bacterial growth during β-lactam therapy. These findings show that *K. pneumoniae* insensitivity to these antibacterial drugs is mediated through the ESBL signaling pathway, suggesting ESBL as a potential focus for combating resistance to β-lactam antibiotics [105].

Nonetheless, it has been determined that PBPs 1, 2, and 3 can still be targeted by certain β-lactam antibiotics such as cefepime, a combination of mecillinam and amoxicillin, piperacillin, aztreonam, and ceftazidime [53,104].

#### 3.3.3. Enzymatic Drug Inactivation

*K. pneumoniae* produces several β-lactamases, with the most prominent being intrinsic narrow-profile Ambler class A β-lactamases, such as TEM-like enzymes and SHV-1, encoded by the *TEM* and *SHV* genes, respectively. These two enzymes mainly confer resistance to penicillins and cephalosporins. Moreover, due to evolution, these enzymes have been developed to ESBLs and are able to destroy aztreonam and oxyimino-β-lactams, e.g., third- and fourth-generation cephalosporins. The genes encoding ESBLs are located on MGEs and are transmitted among different gram-negative bacteria via horizontal gene transfer [43,92,99].

Other class A ESBLs involved in the antimicrobial resistance of *K. pneumoniae* are enzymes from the CTX-M, GES, PER, and VEB families, with VEB standing for Vietnam extended-spectrum β-lactamase [43]. However, the most critical mechanism of β-lactam resistance in *K. pneumoniae* is carbapenem resistance, primarily mediated by serine carbapenemases from class A KPC, MBLs from class B (e.g., NDM, VIM, and IMP), and OXA-type carbapenemases from class D [43,92]. Genes responsible for carbapenemase production are predominantly plasmid-borne and disseminate through horizontal gene transfer [99]. It has been found that β-lactamases KPC-2 and KPC-3 are involved in outbreaks of *K. pneumoniae*-related diseases worldwide [106].

Carbapenem resistance related to KPCs in vivo is associated with alterations in the *bla_KPC_* gene. Examples include mutations in the *bla_KPC_* gene, acquisition of *bla_KPC-2_*-harboring plasmids, mutations and reversions in *bla_KPC_* genes, *bla_KPC-2_* duplication, increased gene expression of mutated *bla_KPC_*, and various alterations in the *bla_KPC-3_* gene primarily leading to the bacterium’s insensitivity to the ceftazidime-avibactam combination [100,107].

NDM-1, belonging to the MBLs, contributes to the insensitivity to nearly all β-lactam antibiotics except aztreonam, as well as rendering the new semi-synthetic aminoglycoside plazomicin ineffective. Moreover, bacteria acquiring this enzyme are linked to MDR strains, with *K. pneumoniae* serving as a primary source for the spread of NDM-1 [43].

Enzymes such as the class D β-lactamases OXA-48, OXA-51, OXA-181, and OXA-237, along with the most prominent ESBLs, including the classical oxacillinases OXA-11 and OXA-15, are involved in the resistance of this pathogen to antibacterial agents. It has been recently established that strains producing OXA-48, encoded by blaOXA-48 genes, are often recognized as a source of nosocomial infections in Middle Eastern and European in-patient settings [43]. Additionally, extensive genetic variation in *bla_OXA-48_*-like-bearing plasmids has been detected in *K. pneumoniae* [108]. However, these β-lactamases are highly effective at breaking down carbapenems, while they exhibit low hydrolytic activity against clavulanic acid [43]. Genes such as *bla_GES_*, *bla_SFO_*, *bla_PER_*, *bla_TLA_*, *bla_VEB_*, and *bla_KLUC-5_*, which encode other class A ESBLs, are less commonly found [97].

An enzyme, AmpC from the class C β-lactamases, evolved in *K. pneumoniae* through the translocation of the *AmpC* gene from the chromosome to a plasmid. This enzyme hydrolyzes extended-spectrum β-lactams and is resistant to the suppressive effects of β-lactamase inhibitors, including clavulanic acid. However, it does not have the capability to degrade carbapenems. Concluding, due to the activity of various β-lactamases, *K. pneumoniae* exhibits resistance to carbapenems, penicillins, cephalosporins, and monobactams [99].

One of the mechanisms used by *K. pneumoniae* to confer resistance to aminoglycosides is through the action of AMEs, primarily AAC(6′)-Ib, and enzymes from the AAC(3)-II subclass, such as AAC(3)-IIa, AAC(3)-IIb, and AAC(3)-IIc. Among these, AAC(3)-IIa is the most commonly found in this bacterium. The genes that encode these enzymes are present on plasmids and transposons, facilitating their easy transfer between strains. It has been established that AAC(6′)-Ib is frequently found in strains possessing ESBL genes on the same plasmid, while the enzyme AAC(3)-IIa is typically detected in strains with the blaCTX-M gene. The enzyme AAC(6′)-Ib inactivates aminoglycosides like tobramycin and amikacin, but not gentamicin, whereas AAC(3)-II enzymes lead to insensitivity to gentamicin, netilmicin, and tobramycin [43,95,99].

Furthermore, the PMQR *aac(6*′*)-Ib-cr* gene is the only gene encoding an AAC that contributes to decreased quinolone efficacy through the *N*-acetylation of their piperazinyl amine group in *K. pneumoniae* [97,109]. This enzyme is capable of inactivating limited-spectrum quinolones like norfloxacin and ciprofloxacin, which have an unsubstituted piperazine group, the substrate for this enzyme. Originally found on plasmids in *K. pneumoniae*, this gene has also been recently discovered on chromosomes. Thus, insensitivity to aminoglycoside antibiotics is frequently accompanied by insensitivity to β-lactam agents and DNA gyrase inhibitors. The gene *aac(6*′*)-Ib-cr* contributes to resistance against antibacterial agents such as aminoglycosides like kanamycin, tobramycin, and amikacin, as well as the aforementioned quinolones [97].

#### 3.3.4. Efflux Pumps

The most predominant efflux systems among efflux pumps in this pathogen are AcrAB-TolC and OqxAB, both from the RND family. The AcrAB-TolC system is composed of the membrane fusion protein AcrA, the efflux transporter AcrB, and the OM channel protein TolC. Various factors, including the AcrA, RamA, and RamR proteins, participate in the regulation of AcrAB-TolC expression. Alterations in the genes encoding these regulatory factors can contribute to increased expression of this pump, leading to an insensitivity to β-lactams, macrolides, fluoroquinolones, and tetracyclines [100,110]. In addition to these efflux pumps, tigecycline insensitivity in this bacterium also involves members of the SMR and MFS superfamilies, such as KpnEF and the MFS member Tet(A) [97,100].

Additionally, antimicrobial resistance to aminoglycosides in *K. pneumoniae* is complex, with changes in cell permeability linked to modifications in the KpnEF and AcrAB-TolC efflux pumps, as well as the potential absence of the KpnO porin. These alterations contribute to varying degrees of insensitivity to gentamicin, tobramycin, streptomycin, and spectinomycin. Additionally, these efflux systems play a role in resistance to polymyxin [97].

The genes encoding the OqxAB drug efflux system, named *oqxAB*, are typically plasmid-encoded and primarily regulated by the RarA protein, with additional regulation by RamA. Overexpression of *rarA* is associated with the MDR phenotypes of *K. pneumoniae*. When the *oqxAB* genes are transferred to a plasmid, it can lead to a tenfold increase in efflux pump manifestation, resulting in a significant reduction in the efficacy of various antimicrobial agents, including fluoroquinolones, chloramphenicol, quinoxaline compounds, and trimethoprim, thereby contributing to a fourfold or greater increase in drug resistance development [99,111].

In conclusion, efflux pumps in *K. pneumoniae* are responsible for resistance to tetracyclines, quinolones, fluoroquinolones, nitrofurantoin, chloramphenicol, polymyxins, macrolides, aminoglycosides, cephalosporins, chlorhexidine, rifampin, and some biocides [99].

### 3.4. Enterobacter *spp.*

MDR *E. aerogenes* and *E. cloacae* have emerged as significant threats over the past 35 years, particularly in causing nosocomial opportunistic infections in immunocompromised patients [2,27]. The bacterium *E. aerogenes* has recently been redefined as *K. aerogenes* due to its genetic similarity to the genus *Klebsiella*. The spread of *Enterobacter* spp. is facilitated by complementary regulatory pathways that effectively control membrane integrity, thereby providing bacterial defense and promoting the activity of detoxifying enzymes responsible for antibiotic degradation or neutralization [2,112].

Moreover, these bacterial species can obtain numerous MGEs that significantly contribute to resistance against antimicrobial agents [2,112]. Thus, *Enterobacter* spp. exhibit intrinsic insensitivity to aminopenicillins, such as ampicillin, and extended-spectrum cephalosporins. Additionally, through the possession of MGEs, they demonstrate insensitivity to many antibiotics, including third-generation cephalosporins and carbapenems, complicating the choice of an appropriate therapy [113].

#### 3.4.1. Drug Uptake Limitation

The main OM proteins that significantly contribute to AMR in *Enterobacter* spp. have been identified as the predominant porins OmpE35, OmpE36, and OmpE37 in *E. cloacae*, and Omp35, Omp36, and Omp37 in *K. aerogenes*. Additionally, both strains share the porins LamB and PhoE, which also play roles in resistance mechanisms [114].

During antibacterial treatment, it has been observed that certain *Enterobacter* spp. sequentially modify the expression of various porins. For example, the Omp35 porin may be substituted with Omp36, which features a smaller channel size. Consequently, strains lacking Omp35, but producing Omp36 generally exhibit intermediate susceptibility to carbapenems, whereas strains lacking both porins demonstrate carbapenem resistance. Additionally, the overproduction of the LamB porin, along with strains exhibiting a lack of porin expression or decreased porin functionality, can further decrease β-lactam susceptibility. Mutations that cause structural alterations in the lumen eyelet region of *K. aerogenes* Omp36 have also been reported, leading to reduced β-lactam permeability [27].

Interestingly, a small protein called OmpX, found in the OM, regulates the synthesis of Omp35. Overproduction of OmpX results in a decreased expression of Omp35. Consequently, the reduced production, loss, or mutations of porins leads to a decreased OM permeability in *Enterobacter* spp. [112].

#### 3.4.2. Drug Target Modification

Alterations in the OM can modify LPSs, further reducing the susceptibility of *Enterobacter* spp. to polymyxins [114]. Many various genes and operons are essential in modifying LPSs in *Enterobacter* spp., significantly influencing their susceptibility to antimicrobial agents. These genes encode enzymes directly involved in LPS modification. For instance, the *pmrA/pmrB* genes modify lipid A through the *arnBCADTEF* operon, alongside the *pmrC* and *pmrE* genes [115].

Additionally, genes like *phoP/phoQ* contribute by activating the *pmrHFIJKLM* operon and inducing the PmrAB TCRS through the *pmrD* gene. The *arnBCADTEF* operon is pivotal in lipid A modification, adding pEtN and L-4AraN [115]. In polymyxin-resistant bacteria, alterations in the *phoQ*, *pmrB*, and *pmrA* genes have been detected. Plasmid-mediated genes, such as *mcr-1*, *mcr-4*, and *mcr-5*, which encode pEtN transferases, also contribute to LPS modifications and polymyxin resistance in *Enterobacter* spp. These genetic mechanisms are essential for LPS structure alteration and confer resistance to polymyxins [114,115].

However, resistance mechanisms associated with drug target modification are mainly related to quinolones. In *Enterobacter* spp., mutations in the QRDRs of targeted enzymes, such as DNA gyrase and topoisomerase IV, lead to high-level insensitivity, primarily through point mutations in chromosomal genes like *gyrA*, *gyrB*, *parC*, and *parE*. PMQR is mediated by QnrA, QnrB, and QnrS, which belong to the Qnr family. These proteins shield bacterial enzymes, DNA gyrase, and topoisomerase IV, from the lethal effects of quinolones. These mechanisms have also been identified alongside genes encoding ESBLs and AmpC on the same plasmid, or through other ways. In *E. cloacae*, resistance to aminoglycosides is facilitated by genes such as *armA* and *rmtB* that encode 16S rRNA methyltransferases [27,114,116].

#### 3.4.3. Enzymatic Drug Inactivation

The synthesis of β-lactamases is the primary process underlying β-lactam insensitivity. Both *K. aerogenes* and *E. cloacae*, in particular, possess a significant ability to regulate these resistance pathways. Typically, *Enterobacter* spp. produces low levels of cephalosporinase corresponding to chromosomal AmpC β-lactamase, which contributes to resistance to first-generation cephalosporins. However, resistance based on chromosomal acquisition can lead to a marked increase in the synthesis of AmpC cephalosporinase, especially upon exposure to subinhibitory concentrations of carbapenems [112,114].

The overproduction of AmpC β-lactamase, resulting from the loss of AmpR function or the acquisition of plasmid-encoded ampC, contributes to resistance against third-generation cephalosporins. *K. aerogenes* can also integrate large plasmids carrying the ampC gene, such as *bla_CMY-10_*, which typically originates from the chromosome. Without the selective influence of antibiotics, this form of genetic material transfer can facilitate the widespread dissemination of resistance mechanisms. This AmpC-related resistance is often linked to the expression of ESBLs. *K. aerogenes* primarily produces TEM-type β-lactamases and those of the CTX-M family, particularly TEM-24 and CTX-M-2 [112,114].

In *E. cloacae*, a variety of ESBLs, including TEM, SHV, and CTX-M types, have been identified. Carbapenemases, such as NDM and VIM types, have been detected in both *E. aerogenes* and *E. cloacae*. Additionally, certain KPC-type or class D β-lactamases, such as OXA-48, which demonstrate carbapenemase activity, have been reported. Recently, MBLs, including IMP-type enzymes, NDM-, GIM-, and VIM-type carbapenemases, as well as serine carbapenemases like KPC and FRI (French imipenemase), have been identified. Among these, the OXA-48-type serine carbapenemase is the most prevalent [112,114,117]. Furthermore, the primary AMEs—AAC(3)-IIa, AAC(6′)-Ib, and ANT(2″)-I—encoded by the *aac(3)-IIa*, *aac(6*′*)-Ib*, and *ant(2*″*)-Ia* genes, respectively, have also been detected [112,114].

Clinical strains often exhibit multiple enzymes. Enzymatic insensitivity to fluoroquinolones has also been described due to the presence of a two-point mutation allele of *aac(6*′*)-Ib*, known as the *aac(6*′*)-Ib-cr* gene, which enables acetylation of ciprofloxacin and norfloxacin. This resistance mechanism spreads rapidly through its association with the *blaOXA-1* gene on various MGEs [112,114].

#### 3.4.4. Efflux Pumps

The primary efflux mechanisms responsible for the extrusion of antimicrobial drugs, including fluoroquinolones, tetracycline, and chloramphenicol, involve the overproduction of the AcrAB-TolC pump, along with efflux proteins such as EefC and AcrZ [27]. Homologues of OqxAB have also been detected in some *Enterobacter* spp. [27]. The main genes encoding drug efflux pumps for fluoroquinolones are *qepA*, *acrAB-tolC*, *oqxAB*, *sugE*, and *emmdR*, while for aminoglycosides it is *acrAB-tolC* [114]. MarA, SoxS, and RamA are transcriptional regulators that trigger AMR by activating efflux pumps [112].

The summarizing data of AMR mechanisms is provided in Table 1.

## 4. High Risk Clones of Gram-Negative ESKAPE Bacteria

The term “clone” describes isolates from different sources, locations, or times that exhibit extensive phenotypic and genotypic similarities, indicating a common origin. This definition is often applied to multidrug-resistant bacteria found in various locations [118,119].

The definition of “high-risk clone” refers to pathogen clones that play a significant role in the rapid spread of antibiotic resistance. These clones are alarming because they complicate treatment and efficiently spread MGEs carrying AMR genes [119,120,121].

In ESKAPE pathogens, high-risk clones play a substantial role in the worldwide dissemination of resistance mechanisms [18]. For instance, some carbapenem-resistant strains, such as *A. baumannii*, *P. aeruginosa*, and certain *Enterobacterales*, are linked to increased patient mortality and pose significant therapeutic challenges [122].

The global spread of MDR bacteria is linked to specific lineages: *A. baumannii* is classified into “international high-risk clones” (ICs), *K. pneumoniae* into clonal groups (CGs) defined by core-genome multilocus sequence typing (cgMLST) and sequence types (STs), while *Enterobacter cloacae* complex and *P. aeruginosa* are categorized primarily by STs (Table 2) [123,124,125,126].

Genomic analyses provide essential insights into the genetic basis of AMR in high-risk clones of *A. baumannii*, *K. pneumoniae*, *P. aeruginosa*, and *Enterobacter* spp. These analyses help uncover the complex clonal structures, resistance determinants, and virulence factors of these pathogens. Surveillance of these high-risk clones is critical for the prompt identification of new insensitivity patterns and for tracking the dissemination of resistance genes. Timely identification of emerging clones enables targeted control strategies, helping to maintain the effectiveness of existing antibiotics and enhance responses to AMR at the public health level [121,137,138].

## 5. Upcoming Novel Treatment Strategies to Combat AMR Resistance of Gram-Negative ESKAPE Bacteria

**Figure 5:**
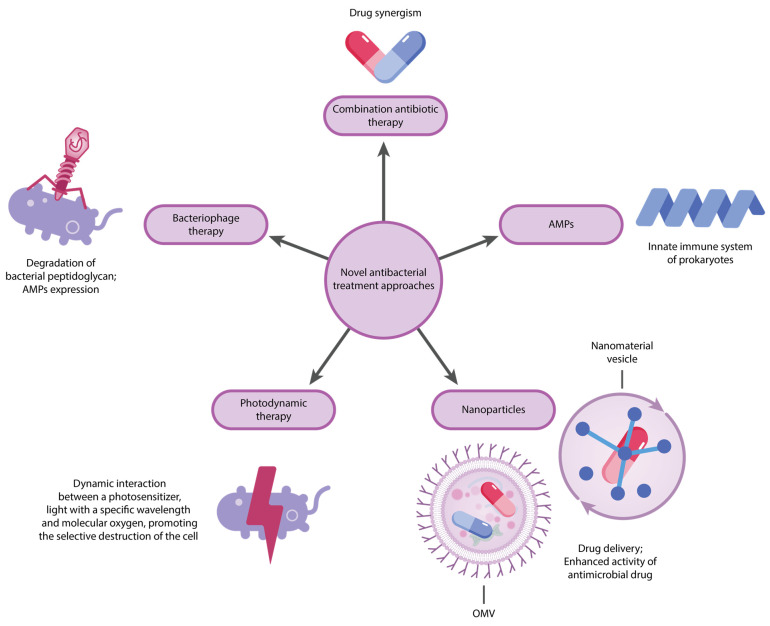
Innovative antimicrobial strategies and their mechanisms of action (adapted and reused from the source [141] under the condition of Creative Common CC BY license, also supplemented on the basis of data from sources [14,36,142]).

The treatment of infections generally relies on antibacterial agents, used alone or in polytherapy. However, the efficacy of these agents against ESKAPE pathogens continues to decline annually, raising concerns about the future due to the restricted use of effective therapeutic alternatives. A review of the Clinical and Laboratory Standards Institute (CLSI) guidelines reveals that, since 2010, numerous antibacterial agents effective against ESKAPE pathogens have been removed, with only a few new antibiotics or combinations introduced. This underscores the critical necessity to investigate alternative treatment strategies to manage diseases primarily triggered by ESKAPE pathogens [139]. To address this challenge, novel treatment approaches are being intensively studied, including combination antibiotic therapy, antimicrobial peptide (AMP) therapy, bacteriophage therapy, nanoparticles [140,141], including OMVs [14,36,142], and photodynamic therapy (Figure 5) [140,141,143]. These approaches seek to counteract resistance mechanisms and enhance the effectiveness of treatment options [140].

An example of antibacterial combination therapy includes the use of colistin and tigecycline in conjunction with other antibacterial drugs to treat infectious diseases caused by *K. pneumoniae* and *A. baumannii* infections [139,140]. Another promising combination involves vaborbactam, a beta-lactamase inhibitor, which helps restore meropenem’s efficacy by inhibiting *K. pneumoniae* carbapenemases [140,144]. Beta-lactamases can be suppressed by metal chelators such as ethylenediaminetetraacetic acid (EDTA), deferasirox, and deferoxamine. These substances are essential for the enzymes’ function [140,145,146,147,148]. Studies have demonstrated that combining certain antibacterial drugs, including imipenem, tobramycin, and vancomycin, with these chelating substances results in a reduction in bacterial burden in *S. aureus*, *P. aeruginosa*, and *E. coli* in mouse models [139]. Quorum quenchers are molecules that inhibit quorum sensing, thus preventing biofilm formation. Among these, 1-[(2,4-Dichlorophenethyl)amino]-3-Phenoxypropan-2-ol has emerged as one of the most promising microbicidal agents. It has been demonstrated to target both dormant cells of P. aeruginosa and non-dormant cells of all ESKAPE pathogens. Furthermore, it has shown the ability to eradicate both planktonic and biofilm-forming antibiotic-insensitive strains [140,149,150]. In combination with various antibiotics, this compound has proven effective against all ESKAPE pathogens, establishing it as a valuable adjuvant in antimicrobial therapy [140,151].

Bacteriophages are natural viruses found in various environmental sources that specifically target bacteria. While they are considered a novel therapeutic option, bacteriophage therapy has been explored since the 1920s. Phages are attractive for several reasons, including their ability to disrupt biofilms, their minimal impact on the commensal microbiota, and their efficacy in eliminating MDR bacteria. These characteristics make them a potential substitute for conventional antibacterial drugs in the fight against resistant infections [152,153]. In a study on bacteriophage therapy, a cocktail of bacteriophages (ECP311, KPP235, and ELP140) delivered through the last left proleg of *Galleria mellonella* resulted in a 100% survival rate of larvae infected with *K. pneumoniae* (KP235). However, multiple doses of bacteriophages were required for effective therapy in vivo [140,154,155]. Similarly, bacteriophage TSK1 was shown to reduce *K. pneumoniae* biofilm by 99–100% in vitro through the release of a depolymerase enzyme that targets the capsular polysaccharides within the biofilm [140,156]. Further studies on *A. baumannii* showed that local application of the virus vBGEC_Ab-M-G7 to wounds led to complete destruction of the bacteria in a rat wound infection model [140,157]. Additionally, the bacteriophage Bφ-C62 exhibited a multiplicity of infection-dependent efficacy against *A. baumannii* in both in vitro and in vivo settings [140,158]. Intranasal administration of the bacteriophage vB_AbaM-IMEAB2, along with intraperitoneal delivery of bacteriophage φkm18p, shielded mice from *A. baumannii* infection, reducing inflammation and decreasing levels of pro-inflammatory cytokines, such as TNF-α and IL-6 [140,159].

AMPs are natural bioactive molecules produced by insects, plants, and humans as part of their immune defense mechanisms against pathogens [152,160]. Given their strong ability to combat bacteria, AMPs are being investigated as potential treatments for bacterial infections [152,161]. These peptides are positively charged and bind to the negatively charged components of bacterial membranes, causing membrane disruption and resulting in bacterial cell death [17,152]. In addition to their membrane activity, certain AMPs can penetrate bacterial cells and interfere with vital intracellular functions, such as DNA and RNA synthesis, further contributing to their antimicrobial effects [17,152].

Evidence suggests that many AMPs exhibit significant efficacy against ESKAPE pathogens. For instance, the AMP S-thanatin and its analogs have shown enhanced antimicrobial effects against *K. pneumoniae* in both in vitro and in vivo models. S-thanatin displayed MIC values 2- to 8-fold lower than those of its analogs, demonstrating superior potency [140,162]. Similarly, Feleucin-K3 has exhibited strong activity against *P. aeruginosa*, including multidrug-resistant clinical strains [140,163].

Other AMP lactoferrin and its derived peptides have demonstrated antibacterial activity against *E. coli*, *S. aureus*, *Acinetobacter* spp., and *P. aeruginosa* in murine models [140,164]. Thus, these findings highlight the capability of AMPs as innovative therapeutic agents to combat resistant bacterial diseases [140,165].

Currently, nanomaterials, such as nanoparticles and nano-drug delivery systems, have shown significant promise in managing infections, particularly those caused by MDR pathogens. Nanoparticles enhance treatment effectiveness through two main mechanisms: enhancing the action of existing antibiotics and introducing novel bactericidal activities independent of conventional drugs [141,166,167].

By functioning as drug carriers, nanomaterials can bypass cellular barriers to deliver antibiotics directly to the bacterial cytoplasm. This targeted delivery allows for controlled drug release, reducing the required dosage, minimizing side effects, and improving the pharmacokinetics, therapeutic efficacy, and cost-effectiveness of treatments. These advancements hold significant potential in combating antibiotic resistance globally [141,166,167].

It has been established that zinc oxide-based nanoparticles exhibit notable antibacterial efficacy against a range of gram-negative pathogens, such as *E. coli* and *P. aeruginosa*, and gram-positive pathogens like *B. subtilis* and *S. aureus* [141,168]. Furthermore, one study has shown that chemically synthesized zinc oxide nanoparticles are effective in suppressing the growth of carbapenem-resistant *A. baumannii* strains [141,169].

Recently, significant attention has been directed towards OMVs, which are projected to be essential in drug delivery systems. These vesicles are being explored for their potential in transporting antibiotic molecules and for use in vaccine development. Their versatility and biocompatibility position them as strong candidates for enhancing the efficacy of various treatments, especially in overcoming challenges posed by antibiotic resistance and improving vaccine delivery. The growing interest in OMVs suggests a promising future in both therapeutic and preventive medicine [14,36,142].

Photodynamic therapy relies on the interaction between a photosensitizer, light of a specific wavelength, and molecular oxygen, which together enable the targeted destruction of microbial cells. The photosensitizer absorbs light, becoming activated and transferring energy to molecular oxygen, thereby generating reactive oxygen species (ROS). These ROS damage cellular components such as lipids, proteins, and nucleic acids, leading to microbial cell death [140,143].

The efficacy of antimicrobial photodynamic therapy (aPDT) is generally influenced by the type and quantity of photosensitizer, as well as the bacterial classification, particularly whether the bacteria are gram-positive or gram-negative [140,170,171,172]. For example, one study evaluated the effectiveness of three substances, acting as photosensitizers, including Azure A, Toluidine Blue O, and New Methylene Blue, on the gram-positive *E. faecalis* and gram-negative *K. pneumoniae*. It was found that photoinactivation was significantly higher in *E. faecalis* than in *K. pneumoniae*, with an 8 log reduction in *E. faecalis* bacterial count after 80 s of irradiation, compared to a 3 log reduction in *K. pneumoniae* after 300 s. Additionally, a higher amount of photosensitizer adhered to *E. faecalis* compared to *K. pneumoniae*. This suggests that the potency of aPDT therapy depends on the quantity of photosensitizer that attaches to the bacterial cell membrane [140,173]

Another study demonstrated that gram-positive bacteria, like MRSA and *E. faecalis*, exhibited greater sensitivity to aPDT than gram-negative bacteria, such as *P. aeruginosa* and ESBL-producing *K. pneumoniae* [140,174]. The sensitivity varied with the duration and intensity of irradiation, with gram-positive bacteria showing greater reductions in colony counts, particularly *MRSA*, where colonies were reduced by 2 log CFU per ml after just 30 s of irradiation. However, the ESBL-producing *K. pneumoniae* strain showed no significant reduction under any of the irradiation conditions. The difference in efficacy between gram-positive and gram-negative pathogens can be attributed to the structural variations in their cell walls. The cell wall in gram-positive bacteria is simpler and more abundant in peptidoglycan and teichoic acids, which makes these pathogens more vulnerable to aPDT. Gram-negative bacteria, on the other hand, have an additional outer membrane (OM) composed of LPSs, lipoproteins, and negatively charged proteins, which form a barrier that limits the penetration of photosensitizers and impedes the photo-oxidation process. These findings highlight the impact of bacterial cell wall structure on the efficacy of antimicrobial photodynamic therapy, with gram-negative bacteria proving more resistant due to their additional protective layers [140,174,175,176,177].

With the increasing need for new antibacterial drugs, there is a growing emphasis on alternative technologies such as AMPs and aPDT in antibacterial research [140]. Notable progress has been made with bacteriophages, synthetic nanomaterials, and natural nanoparticles [140,141,142]. These strategies show great promise in combating multidrug-resistant bacteria and offer innovative solutions for both healthcare applications and industrial use [140,141].

## 6. Concluding Remarks

The mechanisms of AMR in gram-negative ESKAPE bacteria, such as *K. pneumoniae*, *A. baumannii*, *P. aeruginosa*, and *Enterobacter* spp., underscore the sophisticated strategies these pathogens employ to evade antimicrobial treatments. AMR in these bacteria is driven by various mechanisms, including the generation of anti-biotic-degrading enzymes, alterations in drug binding sites, and the increased efflux of antibacterial drugs. These adaptations significantly diminish the effectiveness of current treatments, making diseases caused by gram-negative ESKAPE bacteria increasingly challenging to manage.

High-risk clones emphasize the critical importance of global cooperation to combat the spread of resistance mechanisms. Continued monitoring and study of these clones are essential to mitigating their impact on healthcare and maintaining the effectiveness of existing antibacterial drugs.

To combat these formidable pathogens, it is crucial to explore and develop novel therapeutic strategies. Recent advances include the use of bacteriophages, which specifically target and kill bacteria, and nanoparticles, which can be engineered to deliver antibiotics more effectively or disrupt bacterial defenses. These innovative approaches, along with the development of new antimicrobial agents, offer promising solutions for overcoming resistance mechanisms.

Equally important in addressing AMR is antimicrobial stewardship. Stewardship programs promote the responsible administration and use of antibacterial drugs, ensuring their prescription only when necessary and at appropriate doses. By optimizing antibiotic use, stewardship efforts help to slow the development of resistance and preserve the effectiveness of current treatments, reducing the overall burden of AMR.

Addressing AMR in gram-negative ESKAPE bacteria requires a multifaceted and innovative approach. By continuing to explore and integrate these new strategies, significant progress can be made in alleviating the global burden of drug-resistant infections and improving treatment outcomes.

## Figures and Tables

**Table 1:** The mechanisms of AMR and their major contributors in gram-negative ESKAPE bacteria.

Mechanisms of AMR	*P. aeruginosa*	*K. pneumoniae*	*A. baumannii*	*Enterobacter* spp.
Drug uptake limitation	OprD, OprH, OprF	OmpK35, OmpK36, OmpK37, OmpK38, OmpK26, PhoE	OmpA, CarO	OmpE35, OmpE36, and OmpE37 in *E. cloacae*; Omp35, Omp36, Omp37 in *K. aerogenes*; LamB and PhoE in both strains
Drug target modification	Methylation of 16S ribosomal RNA (rRNA) by RNA methylases encoded by the *rmtA* and *rmtB* genes; Alterations in the motifs of the *gyrA* and *gyrB* genes within the quinolone-resistant determining region (QRDR motif) in DNA gyrase; Alterations in topoisomerase IV via point mutations of *parC* and *parE* genes; Modification of PBPs, including PBP3, PBP4, and PBP5; Alteration of LPs via of lipid A remodeling; PBP3 is linked to resistance to cephalosporins and carbapenems, while PBP4—to resistance against penicillins and cephalosporins	Alterations of lipid A via mutations in genes such as *mcr-1*, *pbgP*, *pmrE*, *pmrHFIJKLM*, *phoPQ*, *pmrAB*, *mgrB*, *crrAB*, *lpxM*, *ramA*, and *pagP*; Mutations in the chromosomal genes *gyrA*, *gyrB*, *parC*, and *parE*; Synthesis of proteins QnrS, QnrA, and QnrB that shield DNA gyrase and topoisomerase IV; Production of RNA methylases such as ArmA, RmfH, RmfA, and NmpA; PBPs	Remodeling of lipid A via alterations in the *pmrA* and/or *pmrB* genes, with mutations being more prevalent in *pmrB*; Deprivation of LPS molecules due to alterations in the *lpxA*, *lpxC*, and *lpxD* genes; Mutations of *gyrA* and *gyrB* genes, which are responsible for DNA gyrase and *parC* gene, which is responsible for topoisomerase IV; Production of DNA gyrase and topoisomerase Iv protective proteins QrnA, QrnS, and QrnS; Production of ribosomal protective proteins, such as TetM, TetW, TetO, and TetS; Production of 23S rRNA (adenine(2058)-N(6))-methyltransferases, encoded by the *erm(B)*, *erm(C)*, and *erm(F)* genes, as well as the ABC-F-type ribosomal protection protein Msr(E), encoded by the *msr(E)* gene; Synthesis of trimethoprim-resistant dihydrofolate reductases, including DfrA1, DfrA5, DfrA7, DfrA10, DfrA12, DfrA14, DfrA16, DfrA17, DfrA19, DfrA20, DfrA27, DfrB1; PBP2 is linked to imipenem resistance, whereas PBP3 is associated with insensitivity to meropenem, sulbactam, and cefiderocol.	Alteration of lipid A via mutations in *mcr-1*, *pbgP*, *pmrE*, *pmrHFIJKLM*, *phoPQ*, *pmrAB*, *mgrB*, *crrAB*, *lpxM*, *ramA*, and *pagP* genes; Modification of the enzymes DNA gyrase and topoisomerase IV due to alterations in genes such as *gyrA, gyrB*, *parC*, and *parE*; Synthesis of proteins QnrS, QnrA, and QnrB, which protect DNA gyrase and topoisomerase IV; Alteration of LPS due to action of *mcr-1*, *mcr-4*, and *mcr-5* genes encoding pEtN transferases; Production of 16S rRNA methyltransferases, encoded by *armA* and *rmtB* genes
Enzymatic drug inactivation	AmpC, TEM, PSE, PER, VEB, GES, BEL, PIB-1, GPC-1, CARB, OXA-50, OXA-2, OXA-10, OXA-23, OXA-40, KPC, NDM-1, SPM-1,GIM-1, VIM, IMP; APHs, AACs, and ANTs	TEM, SHV-1, CTX-M, GES, PER, VEB, KPC-3, KPC-2, NDM-1, OXA-48, OXA-51, OXA-181, OXA-237, OXA-11, OXA-15; AACs	OXA-23, OXA-24/40, OXA-40, OXA-51, OXA-58, OXA-143, KPC-2, KPC-3, TEM-1,-2, CTX-M, SHV, PER, IMP, NDM, SIM, VIM; AACs, ANTs; ADP-ribosyltransferases, including, Arr-2, Arr-3, and Arr-4; Macrolide 2′-phosphotransferases, Mph(A) and Mph(E); Glutathione transferases from the FosLL, FosA3, and FosA3/FosA4 families; Tet(X) monooxygenases.	AmpC, TEM, SHV, CTX-M-2, TEM-24, IMP-type, NDM-type, VIM-type, GIM-type, OXA-48-type, KPC, FRI AACs, ANTs
Drug efflux	Mainly from RND family: MexCD-OprJ, MexAB-OprM, MexXY, MexEF-OprN	Mainly from RND family: AcrAB-TolC, OqxAB, KexEF, KexC, KexD, EefABC, and KdE; SMR superfamily member KpnEF; MFS superfamily member Tet(A)	AdeABC, MATE, MFS, RND, SMR, ABC	RND family members AcrAB-TolC and OqxAB

**Table 2:** The most prevalent high-risk clones of ESKAPE gram-negative bacteria.

Pathogen	Clonal Classification	Notes
*A. baumannii*	Spread worldwide: CC1P/CC109O, CC2P/CC92O, CC3P/CC187O, CC1P/CC109O (spread worlwide); Common in Europe and North America: CC2P/CC92O, CC3 P/CC187O.	Have caused outbreaks and persisted in ICUs globally for extended periods; Recently, ST136O and ST208O, which are part of CC2P/CC92O, as well as ST758O and ST1054O, which are part of CC636, have been reported; Sequence types ST58O and ST1054O represent a high-risk clone associated with multi-drug resistance; ST20O and ST136O are part of the most predominant and globally disseminated complex, linked to isolates with the capacity to acquire resistance.
*K. pneumoniae*	CG258 (ST258, ST11, ST512), CG14 (ST14, ST51). Recently identified non-clonal group 258 ST307 and ST147	*K. pneumoniae* strain ST258 is the dominant clone*,* mainly associated with the production of KPC-2 and KPC-3 carbapenemases; Additionally, ST11, ST340, and ST512 are single-locus variants of ST258, all of which carry arbapenemase genes; ST11, in particular, is genetically similar to ST258 and linked to the production of KPC, NDM, VIM, IMP, and OXA-48 carbapenemases; Strains of *K. pneumoniae* ST14 and ST15 are frequently producers of ESBLs or carbapenemases, which contribute to antibiotic insensitivity and hospital outbreaks globally; The ST307 and ST147 clones are believed to have originated in Europe during the early to mid-1990s and rapidly gained prominence as global pathogens. These clones have since spread across every continent, excluding Antarctica, and have been responsible for numerous global nosocomial outbreaks, including those linked to endoscopy procedures and long-term care facilities
*E. cloacae complex (CREC)*	ST66, ST78, ST108, ST114, ST171, ST105, ST108, ST93, ST90	ST171 and ST78 clones have shown epidemic potential, partly due to their occasional acquisition of plasmid-borne carbapenemases; Clones ST66, ST171, and ST78 are known to produce ESBLs, including CTX-M-15 and CTX-M-9, with 40% of these isolates also carrying the *mcr-9* gene; The presence of blaOXA-48 and blaCTX-M in MDR-resistant, high-risk *E. cloacae* clones is particularly concerning, as *mcr-9* may transfer to other bacteria or mutate, potentially resulting in colistin resistance; Effective surveillance is essential to identify and control these pathogens, which pose serious treatment challenges and risk the spread of resistance; A newly identified ST66 has exhibited the capacity to gain various antibiotic insensitivity genes and employ its secretion systems, highlighting its risk of developing into a high-risk clone.
*P. aeruginosa*	ST235, ST111, ST277, ST244, ST308, ST395	The primary global high-risk MDR/XDR clones are ST175, ST111, and ST235, with ST175 being the predominant, representing 68% of XDR isolates; ST235 possesses the DprA determinant, which facilitates homologous recombination, potentially enhancing its capacity to obtain and retain external resistance traits. In *P. aeruginosa*, resistance-associated mutations have been identified in ST111 and ST235 clones, including GyrA T83I, ParC S87L, and changes in the oprD protein; Compared to the other two high-risk clones, ST235 (serotype O11) is notably more prevalent and has been identified on all five continents; Similarly, ST111 (serotype O12) is widespread, though not reported in Oceania; In contrast, ST175 (serotype O4) is predominantly found in Europe.

Prepared on the basis of data from the sources [18,125,127,128,129,130,131,132,133,134,135,136].

## Data Availability

No new data were created or analyzed in this study.

## Abbreviations

AAC	aminoglycoside N-acetyltransferase
ABC	ATP-binding cassette
ADC	*Acinetobacter-*derived cephalosporinase
AME	aminoglycoside-modifying enzyme
AMP	adenosine monophosphate
AMPs	antimicrobial peptides
AMR	antimicrobial resistance
anhMP	anhydromuropeptide
ANTs	aminoglycoside O-nucleotidyltransferases
aPDT	antimicrobial pototdynamic therapy
APHs	aminoglycoside O-phosphotransferases
Ara4N	transfer of 4-amino-4-deoxy-1-arabinose
ATP	adenosine triphosphate
BEL	Belgium extended β-lactamase
CARB	carbenicillin-hydrolyzing β-lactamase
CFU	colony-forming unit
cgMLST	core-genome multilocus sequence typing
cKp	classical *K. pneumoniae*
CLSI	Clinical and Laboratory Standards Institute
CRE	carbapenem-resistant enterobacterales
CREC	*E. cloacea* complex
CRKP	carbapenem-resistant *K. pneumoniae*
CTX-M	cefotaxime-hydrolyzing β-lactamase
DNA	deoxyribonucleic acid
EEA	European Economic Area
EDTA	ethylenediaminetetraacetic acid
ESBL	extended-spectrum β-lactamases
ESKAPE	*E. faecium*, *S. aureus*, *K. pneumoniae*, *A. baumannii*, *P. aeruginosa*, and *Enterobacter* spp.
EU	European Union
GES	Guiana extended-spectrum β-lactamase
GPC-1	German *Pseudomonas* carbapenemase
GTP	guanosine triphosphate
hvKp	hypervirulent *K. pneumoniae*
ICs	international high-resistant clones
ICU	intensive care unit
IGs	clonal groups
IM	inner membrane
IMP	imipenemase
IQR	interquartile range
KPC	*K. pneumoniae* carbapenemase
L-Ara4N	4-amino-4-deoxy-L-arabinose
LPS	lipopolysaccharide
M	median
MATE	multidrug and toxic compound extrusion
MBL	metallo-β-lactamase
MDR	multidrug resistance
MFP	membrane fusion or an adaptor protein
MFS	major facilitator superfamily
MGE	mobile genetic element
MIC	minimal inhibitory concentration
MRSA	methicillin-resistant *S. aureus*
NDM	New Delhi metallo-β-lactamase
OM	outer membrane
Omp	outer membrane protein
OMV	outer membrane vesicle
OXA	oxacillinase
PACE	proteobacterial antimicrobial compound efflux
PBP	penicillin-binding protein
PDR	pandrug resistant
PER	*Pseudomonas* extended resistant
PEtN	phosphoethanolamine
PIB-1	*Pseudomonas* imipenem β-lactamase
PMF	proton motive force
PMQR	plasmid-mediated quinolone resistance
PSE	*Pseudomonas*-specific enzyme
QRDR	quinolone-resistant-determinating region
RND	resistance-nodulation-division
ROS	reactive oxygen species
SARS-CoV-2	a strain of corona virus that causes COVID-19
SCO-1	a novel plasmid-mediated class A β-lactamase with carbenicillinase characteristics from *E. coli*
SHV	sulfhydryl reagent variable
SIM	Seoul imipenemase
SMR	small multidrug resistance
SPM	Sao Paulo MBL
STs	sequence types
TCRS	two-component regulatory system
TEM	Temoneira serine β-lactamase of molecular class A
UI	uncertainty interval
US	United States
VAP	ventilator-associated pneumonia
VEB	Vietnam extended β-lactamase
VIM	Verona integron-encoded MBL
VRE	vancomycin-resistant *Enterococcus*
WHO	World Health Organization
XDR	extensively drug-resistant bacteria
Note: The names of genes and bacterial strains are written in italics	
