# Correction: Sakalauskienė et al. Unseen Enemy: Mechanisms of Multidrug Antimicrobial Resistance in Gram-Negative ESKAPE Pathogens. *Antibiotics* 2025, *14*, 63

**DOI:** 10.3390/antibiotics15010022

**Published:** 2025-12-25

**Authors:** Giedrė Valdonė Sakalauskienė, Lina Malcienė, Edgaras Stankevičius, Aurelija Radzevičienė

**Affiliations:** Institute of Physiology and Pharmacology, Faculty of Medicine, Medical Academy, Lithuanian University of Health Sciences, 44307 Kaunas, Lithuania; lina.malciene@lsmu.lt (L.M.); edgaras.stankevicius@lsmu.lt (E.S.); aurelija.radzeviciene@lsmu.lt (A.R.)

**Text Correction**

There was an error in the original publication [1]. The reader has raised a concern regarding misinformation in the abstract section. The correct sentence is as below.

“…driven by the particularly concerning ESKAPE pathogens: the Gram-positive *Enterococcus faecium* and *Staphylococcus aureus*, as well as the Gram-negative *Klebsiella pneumoniae*, *Acinetobacter baumannii*, *Pseudomonas aeruginosa*, and *Enterobacter* spp.”

There are some typos in the published paper.

The word “gram” has been modified to “Gram” in the whole paper.

The word “widespread” has been corrected in Section 1, paragraph 1.

The word “also” has been corrected in Section 3.1.1, paragraph 4, Section 3.2.1, paragraph 3. 

The word “2–3-fold” has been corrected in Section 3.1.1, paragraph 4.

The word “typically” has been corrected in Section 3.1.2, paragraph 4.

The word “be” has been corrected in the last paragraph of Section 3.1.3.

The word ”Resistance” has been removed from the heading of Section 5.

The authors state that the scientific conclusions are unaffected. This correction was approved by the Academic Editor. The original publication has also been updated.
